# Methane oxidation to ethanol by a molecular junction photocatalyst

**DOI:** 10.1038/s41586-025-08630-x

**Published:** 2025-01-20

**Authors:** Jijia Xie, Cong Fu, Matthew G. Quesne, Jian Guo, Chao Wang, Lunqiao Xiong, Christopher D. Windle, Srinivas Gadipelli, Zheng Xiao Guo, Weixin Huang, C. Richard A. Catlow, Junwang Tang

**Affiliations:** 1 Department of Chemical Engineering, University College London, London, UK; 2 State Key Laboratory of Precision and Intelligent Chemistry, iChEM, Key Laboratory of Surface and Interface Chemistry and Energy Catalysis of Anhui Higher Education Institutes, Department of Chemical Physics, University of Science and Technology of China, Hefei, China; 3 School of Chemistry, University of Cardiff, Cardiff, UK; 4 School of Chemistry, University of Leeds, Leeds, UK; 5 Department of Chemistry, University College London, London, UK; 6 Industrial Catalysis Center, Department of Chemical Engineering, Tsinghua University, Beijing, China; 7 Department of Chemistry, The University of Hong Kong, Hong Kong SAR, China

**Keywords:** Photocatalysis, Natural gas, Catalytic mechanisms

## Abstract

Methane, the main component of natural and shale gas, is a significant carbon source for chemical synthesis. The direct partial oxidation of methane to liquid oxygenates under mild conditions^1–3^ is an attractive pathway, but the inertness of the molecule makes it challenging to achieve simultaneously high conversion and high selectivity towards a single target product. This difficulty is amplified when aiming for more valuable products that require C–C coupling^4,5^. Whereas selective partial methane oxidation processes^1–3,6–9^ have thus typically generated C_1_ oxygenates^6,7^, recent reports have documented photocatalytic methane conversion to the C_2_ oxygenate ethanol with low conversions but good-to-high selectivities^4,5,8–12^. Here we show that the intramolecular junction photocatalyst covalent triazine-based framework-1 with alternating benzene and triazine motifs^13,14^ drives methane coupling and oxidation to ethanol with a high selectivity and significantly improved conversion. The heterojunction architecture not only enables efficient and long-lived separation of charges after their generation, but also preferential adsorption of H_2_O and O_2_ to the triazine and benzene units, respectively. This dual-site feature separates C–C coupling to form ethane intermediates from the sites where •OH radicals are formed, thereby avoiding over-oxidation. When loaded with Pt to further boost performance, the molecular heterojunction photocatalyst generates ethanol in a packed-bed flow reactor with greatly improved conversion that results in an apparent quantum efficiency of 9.4%. We anticipate that further developing the ‘intramolecular junction’ approach will deliver efficient and selective catalysts for C–C coupling, pertaining, but not limited, to methane conversion to C_2+_ chemicals.

## Abstract (web summary)

Mechanistic insights into a photocatalyst with an intramolecular junction and two different active sites could inspire further exploration of ‘intramolecular heterojunctions’ as the basis of selective and stable photocatalysts for C–H activation and C–C coupling.

## Main

Selectively photocatalytic conversion of methane to a specific C_2+_ chemical, for example, ethanol, is highly significant for both energy security and low-carbon production of valuable chemicals. However, it remains scientifically challenging to create the necessary C–C coupling microenvironment capable of (1) coordinating the binding of methane molecules, co-reactants and reaction intermediates in close proximity; (2) providing sufficient charge separation and delocalization to drive a specific photo-redox pathway; and (3) facilitating the desorption of the desired product to avoid over-oxidation. Not meeting all these criteria probably explains why the photocatalysts reported to enable selective methane-to-ethanol conversion have achieved only moderate apparent quantum efficiencies (AQE < 0.5)^5,8–10,12^. It is also noted that these conversions use batch reactors, which often accumulate the strong oxidizing species involved, leading to further attack of the product (for example, ethanol), thereby rapidly reducing the selectivity.

**Fig. 1:**
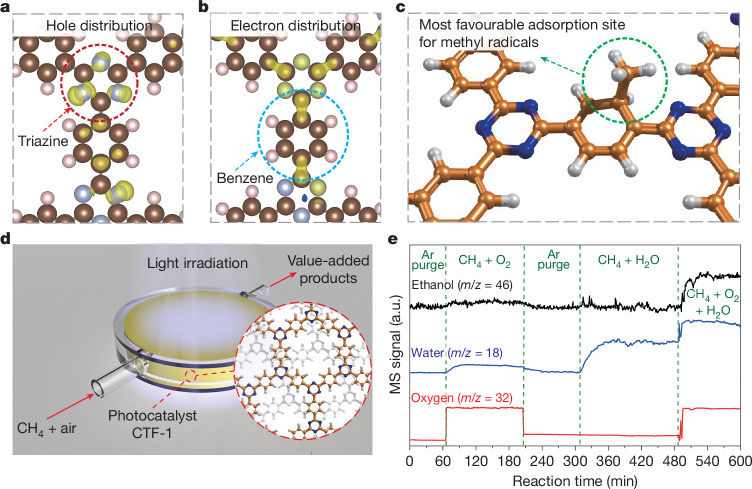
Materials design and experimental set-up. **a**,**b**, Spatial distribution of the HOMO (**a**) and LUMO (**b**) of CTF-1 catalyst. **c**, Most favourable adsorption site of methyl radical on CTF-1 estimated by first-principles calculation (C, N and H atoms are shown in gold, blue and white, respectively). **d**, Schematic representation of the reaction system of the photocatalytic methane oxidation to high-value chemicals; the reactor was sealed by a polytetrafluoroethylene screw-type jacket, the yellow part represents the photocatalyst. **e**, Online MS responses of ethanol, water and oxygen of the outlet gas during the selective photocatalytic oxidation of methane. Reaction conditions, gas flow rate 40 ml min^−1^, ambient temperature and 365-nm LED irradiation. Source Data

With due consideration of the above and the literature, we resorted to a covalent triazine-based framework, covalent triazine-based framework (CTF)-1 (refs. ^13,14^), which contains intrinsic intramolecular heterojunctions formed by alternating triazine and benzene motifs (Fig. 1a–c and Fig. 1d (inset)). To rationalize the electronic and catalytic characteristics of CTF-1, relative to those of C_3_N_4_, density functional theory (DFT) calculations were performed as detailed in the Supplementary Information^15–18^. Analysis of the highest-occupied molecular orbital (HOMO)/lowest-unoccupied molecular orbital (LUMO) bands shows that the triazine motif in CTF-1 can accumulate photo-excited holes (Fig. 1a) that may thus serve as a site for activating methane C–H bonds either directly^6,7^ or indirectly (for example, through •OH radicals generated by photo-holes)^5^ to form methyl radicals. Unlike g-C_3_N_4_, which comprises only triazine motifs and thus requiring dopants or co-catalysts to achieve efficient photocatalysis^5,8–11^, CTF-1 also contains benzene motifs and these enable efficient separation and accumulation of photoelectrons after the initial charge generation step (Fig. 1b). The benzene motif also provides the most exothermic (−116 kJ mol^−1^) binding site for methyl radical species among all binding sites considered (Fig. 1c and Supplementary Figs. 1 and 2). Therefore, such an intrinsic intramolecular heterojunction theoretically facilitates C–C coupling as the newly generated methyl species would spill over to the benzene units after their generation (Fig. 1c), avoiding further oxidation by photo-holes on the triazine units. This encouraging finding prompted the synthesis of CTF-1 by a rapid microwave-assisted process^14^. X-ray diffraction and Raman, Fourier-transform infra-red (FTIR) and solid-state ^13^C nuclear magnetic resonance (NMR) spectroscopy confirm both the polymeric structure and the alternating aromatic units (Supplementary Figs. 3–6)^13,14,19–21^. Scanning electron microscopy shows that the CTF-1 consists of densely packed particles of tens of micrometres (Supplementary Fig. 7).

To identify the effective pathways for methane transformation over CTF-1, two typical reactions, methane partial oxidation (CH_4_ + O_2_) and steam reforming (CH_4_ + H_2_O), were first studied in a packed-bed photocatalytic reactor with continuous flow of the reactant gas under irradiation from a 100-W light-emitting diode (LED) (365 nm) (spectrum in Supplementary Fig. 8) without additional heating or pressurization (Fig. 1d). The activity was first monitored qualitatively by mass spectrometry (MS) (Fig. 1e). Background spectra were collected for up to 60 min under light irradiation and continuous argon flow through the fixed-bed reactor. The methane partial oxidation reaction was then carried out by feeding premixed methane and oxygen to the reactor (spanning 60–200 min in Fig. 1e). A notable amount of ethanol was generated during the reaction (also confirmed by ^1^H NMR in Supplementary Fig. 9), indicating a C–C coupling process of methane on the CTF-1 catalyst. Water was detected as a byproduct, indicating the occurrence of the exothermic overall redox reaction $$2{\rm{C}}{{\rm{H}}}_{4}+{{\rm{O}}}_{2}\to \,$$$${\rm{C}}{{\rm{H}}}_{3}{\rm{C}}{{\rm{H}}}_{2}{\rm{OH}}+{{\rm{H}}}_{2}{\rm{O}}({\Delta }_{r}{G}_{m}^{\theta }(298.15{\rm{K}})=-\,295.51\,{\rm{kJ}}{{\rm{mol}}}^{-1})$$. The gas line was then switched back to argon purging for around 100 min to remove all reactants and products until all signals were stabilized. Subsequently, the steam reforming (CH_4_ + H_2_O) was undertaken by feeding methane with saturated water vapour (spanning 310–490 min in Fig. 1e), but little ethanol was obtained, indicating that the endothermic reaction between methane and water to form ethanol and hydrogen ($$2{\rm{C}}{{\rm{H}}}_{4}+{{\rm{H}}}_{2}{\rm{O}}({\rm{g}})\to {\rm{C}}{{\rm{H}}}_{3}{\rm{C}}{{\rm{H}}}_{2}{\rm{OH}}({\rm{g}})+{{\rm{H}}}_{2}$$, $${\Delta }_{r}{G}_{m}^{\theta }(298.15{\rm{K}})=+\,161.71{\rm{kJ}}{{\rm{mol}}}^{-1}$$) is very sluggish under the experimental conditions. However, when both water and oxygen were present (spanning 490–600 min in Fig 1e), the generation of ethanol increases by a factor of roughly seven compared with that observed in the presence of oxygen alone, indicating that water molecules greatly promote the reaction. Thus, both water and oxygen are deemed crucial in driving the process. Since the overall reaction equation in this period is 2CH_4_ + O_2_ + H_2_O → C_2_H_5_OH + 2H_2_O, a marked enhancement in the water signal was observed, indicating that water was produced as a byproduct. As the reaction continues, the water content remains constant between 490 and 540 min and then reduces by around 5%. This is probably due to the slight adsorption of the generated water on the surface of the highly porous CTF-1 catalyst as discussed in the control experiment (Supplementary Fig. 10) and the in situ diffuse reflectance infrared Fourier-transformed spectroscopy (DRIFTS) analysis (Supplementary Fig. 11).

The photocatalytic transformation of methane was further quantified over a series of runs (Supplementary Tables 1 and 2), illustrating the trade-off between conversion and product selectivity. The methane conversion rate decreases as the concentration of oxygen is reduced while the selectivity towards ethanol is enhanced (Entries 1–4). The methane to oxygen ratio of CH_4_:O_2_ = 16:1 leads to the highest ethanol selectivity of 78.6% with a methane conversion rate of 1.7%. For comparison, this selectivity is comparable with that of methane conversion to C_2_ products by thermocatalysis operated at 600–800 °C, although the thermal catalytic process achieves methane conversions of around 25% (refs. ^22,23^). The conversion and selectivity we achieved are more than ten times higher than the methane-to-methanol conversion and selectivity over a heterogeneous catalyst operated at medium temperature and/or pressure^2^, and comparable with the conversion and selectivity for direct methane conversion to methanol over supported Cu and Rh catalysts at 200–400 °C (refs. ^1,3^). As the CH_4_:O_2_ ratio increases from 4:1 to 32:1, the carbon balance also increases from 81.5% to 94.3% on the basis of the gas chromatography (GC)–flame ionization detection (FID) (GC–FID) analysis. This indicates the presence of minor products (potentially long-chain products or oxygenates) that cannot be detected directly by GC–FID, especially at lower CH_4_:O_2_ ratios. These minor products were then detected by in situ synchrotron radiation photoionization MS. Apart from C_2_H_5_OH, CO_2_ and CH_3_OH, small amounts of CO, CH_3_COOH, C_5_-C_9_ oxygenates and/or C_10_ hydrocarbons are also observed (Supplementary Figs. 12 and 13 and Supplementary Table 3), which contribute to the carbon balance that cannot be detected by GC–FID. The highest methane conversion rate is achieved with a dry gas flow rate (DGFR) of 2,000 ml h^−1^ but drops drastically from 2.5% to 0.1% when aiming for overall higher production rates by increasing DGFR to 10,000 ml h^−1^, whereas the selectivity of the process towards ethanol changes from 78.6% to 61%.

Control experiments assessed the performances of the prototypical inorganic photocatalysts TiO_2_ and polymeric g-C_3_N_4_, respectively. The data in Table 1 confirm^24^ that only CO_2_ is generated by the TiO_2_ photocatalyst (Entry 2). The g-C_3_N_4_ (Entry 3) converts methane to ethanol with a production rate of only 29 μmol h^−1^ and an equal selectivity of 46% methane to methanol and ethanol, similar to the performance reported previously^5,8^ for controls with the unpromoted photocatalyst. This contrasts with CTF-1 (Entry 1), which produces five times more ethanol with a selectivity of 79%. To further improve the activity, the CTF-1 photocatalyst was decorated with platinum species^25^ (Supplementary Fig. 14) and the incorporation of 3 wt% PtO_x_ increases the ethanol production rate by nearly 50% while maintaining the selectivity at approximately 80% under identical reaction conditions (Table 1; Entry 4). Incorporating ruthenium oxide species (Supplementary Fig. 15)^26^, in contrast, decreases both the yield, from 122 to 99 μmol h^−1^, and the ethanol selectivity, from 79% to 72% (Entry 5). Supplementary Table 4 provides an overview of photocatalysts reported to convert methane to ethanol. The systems all reach reasonable-to-high selectivities, with a vacancy-rich g-C_3_N_4_ photocatalyst^11^ standing out for producing ethanol with a selectivity of 85.1% and a record mass-normalized ethanol production rate of 280 μmol h^−1^ g^−1^. The methane conversion achieved with this and the other reported photocatalysts is very low, however, whereas our photocatalysts CTF-1 and PtO_x_-CTF-1 produce significantly more ethanol in absolute terms (μmol h^−1^) while at the same time attaining high amounts of ethanol selectivity and methane conversion. We also note (and discuss further in the note to Supplementary Table 4) that a reliable comparative assessment of photocatalysts developed and evaluated in different laboratories is difficult despite the availability and use of several performance indicators. This is because measured photocatalytic performances (activity, selectivity and also apparent quantum yield) depend sensitively on the experimental set-up and operational conditions used (such as the type and operation of the reactor, the reactant feed rate and ratio, and the nature of the light source used and so on, which typically differ from study to study), highlighting the benefits of and need for community-wide standardization of photocatalytic reactors and measurement protocols.

**Table 1:** Methane oxidation on CTF-1, TiO_2_, g-C_3_N_4_, PtO_x_/CTF-1 and RuO_x_/CTF-1 in a packed-bed flow reactor^a^

Photocatalyst	CH_4_ Conversion, %	Product yield, μmol h^−1^				Product selectivity on the basis of carbon, %				Reactants flow rate, mmol h^−1^			
		C_2_H_5_OH	CH_3_OH	C_2_H_6_	CO_2_	C_2_H_5_OH	CH_3_OH	C_2_H_6_	CO_2_		CH_4_	O_2_	CH_4_/O_2_
CTF-1	1.65 (±0.10)	122.4 (±2.6)	–	4.0 (±1.9)	26.0 (±1.2)	78.6 (±1.7)	–	1.1 (±0.7)	8.2 (±0.5)		18.9 (±0.5)	1.2 (±0.1)	15.8
TiO_2_	0.81 (±0.06)	0	–	–	121.8 (±17.6)	–	–	–	86.8 (±11.6)		19.3 (±0.4)	1.2 (±0.1)	16.1
g-C_3_N_4_	0.61 (±0.16)	29.2 (±0.1)	57.3 (±0.5)	–	2.04 (±0.5)	46.1 (±0.7)	45.67 (±0.4)	–	1.5 (±0.4)		20.7 (±0.7)	1.3 (±0.1)	15.9
3 wt% PtO_x_/CTF-1	2.33 (±0.13)	167.6 (±14.7)	–	2.0 (±1.8)	52.3 (±5.8)	79.6 (±7.0)	–	0.8 (±0.5)	11.8 (±1.2)		17.3 (±0.3)	1.1 (±0.1)	15.7
3 wt% RuO_x_/CTF-1	1.54 (±0.10)	99.2 (±10.3)	–	3.2 (±2.5)	26.0 (±2.5)	72.2 (±7.5)	–	1.4 (±0.9)	9.8 (±1.1)		17.6 (±0.3)	1.1 (±0.1)	16.0

^a^CH_4_ source is 20% CH_4_/Ar and O_2_ source is the humidified simulated air (20% O_2_/N_2_). Values of yield and selectivity are averages during 4-h light irradiation. The error in parenthesis is the calculated s.d. over three samples with each measured three times. The reactant flow rates were obtained from the average value of flow concentrations after pouring for 6 h in the dark.

**Fig. 2:**
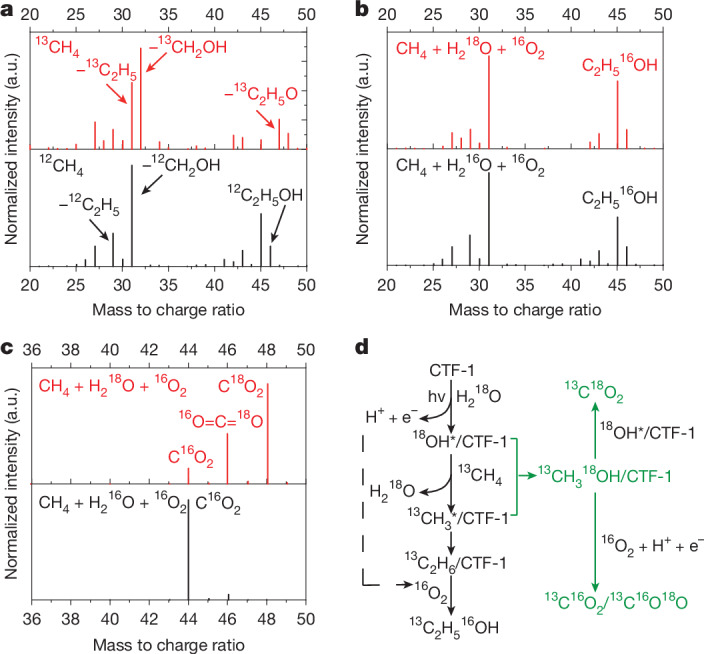
GC–MS spectra of isotope-labelled reactants and products during the photocatalytic selective oxidation of methane over CTF-1. **a**, Mass spectra of the main product ethanol when using ^13^CH_4_ + ^16^O_2_ + H_2_^16^O (top) and ^12^CH_4_ + ^16^O_2_ + H_2_^16^O (bottom) as a feed gas. **b**, Mass spectrum of generated ethanol through ^12^CH_4_ + ^16^O_2_ + H_2_^18^O (top) and ^12^CH_4_ + ^16^O_2_ + H_2_^16^O (bottom). **c**, Mass spectrum of obtained CO_2_ when using ^12^CH_4_ + ^16^O_2_ + H_2_^18^O (top) and ^12^CH_4_ + ^16^O_2_ + H_2_^16^O (bottom) as reactants. **d**, Scheme of proposed reaction pathway for photocatalytic methane oxidation to ethanol by humidified air over CTF-1 catalyst. Source Data

To further clarify the reaction mechanism and the carbon source in the products, isotopic labelling was carried out. ^13^CH_4_ was first used to identify the carbon source for ethanol production. As shown in Fig. 2a, the most significant peak at mass/charge (*m*/*z*) = 31 under ^12^C conditions (Fig. 2a, bottom panel; assigned as ^12^CH_2_OH^+^ fragment ions) shifts to *m*/*z* = 32 (^13^CH_2_OH^+^) when the feed source was switched to ^13^CH_4_ (Fig. 2a, top panel). The second strongest peak at *m*/*z* = 45 represents ^12^CH_3_^12^CH_2_O^+^ shifting to *m*/*z* = 47 (^13^CH_3_^13^CH_2_O^+^). All other peaks also shift to the higher *m*/*z* ratio with constant relative intensities. The commercially available 2-^13^C-ethanol was also analysed, and showed a spectrum identical to that of the isotopically labelled ethanol produced in this work (Supplementary Fig. 16), indicating that ethanol was produced from methane oxidation. More importantly, there is no fragment detected at *m*/*z* = 46 associated with the ^12^CH_3_^12^CH_2_OH^+^ when using ^13^C isotope-labelled methane as a reactant, which strongly suggests that all carbon incorporated in ethanol originates from methane rather than from the polymer photocatalyst. The reaction pathway was investigated by total ion chromatogram, mass spectrum and GC–FID (Supplementary Figs. 17–22;) to indicate that ethane is the reaction intermediate, as discussed further later.

To investigate the function of water, ^18^O-labelled water was used. As shown in Fig. 2b, regardless of the presence of isotope-labelled water, the oxygen atoms in the produced ethanol are identically detected as ^16^O, which should be from the oxidant ^16^O_2_. As the ^18^O exchange between water and oxygen gas was undetectable (Supplementary Fig. 23), the isotopic labelling results indicate that ethanol is generated by a reaction process with photocatalytically reduced O_2_ species. On the other hand, CO_2_ as the over-oxidation product shows a different oxygen source. As shown in Fig. 2c, when using H_2_^18^O to humidify the feed gas, most of the oxygen atoms detected in the CO_2_ are ^18^O atoms. The ratio of C^16^O_2_:^18^O = C = ^16^O:C^18^O_2_ was around 1:3:6. Thus, most of the over-oxidation product CO_2_ should result from the reaction between •OH radicals generated from water and methane molecules, with a minor contribution from O_2_.

This process was further clarified by gas adsorption analysis (Supplementary Fig. 24). The mass change when a mixture of water and methane was used as the feed gas equals the sum of the mass changes when water and methane were added sequentially as the feed gases. Therefore, water does not compete with methane for adsorption on the catalyst. Furthermore, the mass of water adsorbed is nearly twice that of the adsorbed methane, indicating that more water molecules are adsorbed on the polymer photocatalyst than methane (considering that they have similar molar masses). DFT calculations (Supplementary Fig. 25) confirm that when the surface of CTF-1 is hydroxylated by dissociated water, the activation energy of methyl radical formation is greatly reduced (by roughly 186 kJ mol^−1^) compared with the dry surface. Thus, as shown in Fig. 2d, the function of the water is to hydroxylate the catalyst surface through an oxidation reaction with photogenerated holes $({{\rm{H}}}_{2}{\rm{O}}+{{\rm{h}}}^{+}\to \bullet {\rm{OH}}+{{\rm{H}}}^{+})$. The presence of adsorbed •OH radicals facilitates the endothermic C–H bond cleavage of methane and the generation of •CH_3_ radicals, with H_2_O and surface protons as by-products (Supplementary Fig. 25)^2,27^. This contrasts with g-C_3_N_4_-based catalysts, where methane is reported to be activated directly by the photo-holes to generate methanol or ethanol^8,11^. In our system, •CH_3_ radicals couple to form ethane as observed by total ion chromatogram, GC–MS and GC–FID (Supplementary Figs. 17–20). C_2_H_6_ then further reacts with the surface-adsorbed O_2_ to form ethanol and water, as indicated by the isotopic labelling. The kinetic online-mass curves further show that, in the absence of oxygen, a large amount of ethane is generated but only a trace amount of ethanol is detected. When oxygen is added, the amount of ethane decreases, whereas much more ethanol is generated (Supplementary Fig. 20). This indicates that ethane is an intermediate and is converted to ethanol in the presence of O_2_. This is a very different mechanism from the pathway over g-C_3_N_4_-based photocatalysts, where methanol is first formed as the key intermediate to ethanol^8,10–12^. It should be noted that, in the absence of oxygen gas, the water oxidation sites are poisoned because generated protons bind to the nitrogen atom in the triazene ring (Supplementary Fig. 21), and that O_2_ reacts with the protons and thereby regenerates the surface, as also confirmed by DFT calculations (Supplementary Figs. 26 and 41). Finally, the adsorption energies of ethane and ethanol on the CTF-1 surface are similar (Supplementary Table 5), indicating that both can desorb readily after their formation. However, the ready availability of very close surface-adsorbed O-containing species facilitate ethane transformation into ethanol before ethane can desorb. This explains the observations of only a trace amount of ethane and a high selectivity towards ethanol over CTF-1. As a competing reaction, some •CH_3_ radicals may recombine with •OH radicals generated from water to form methanol, which tends to be further oxidized to CO_2_, as indicated by the isotopic measurement.

**Fig. 3:**
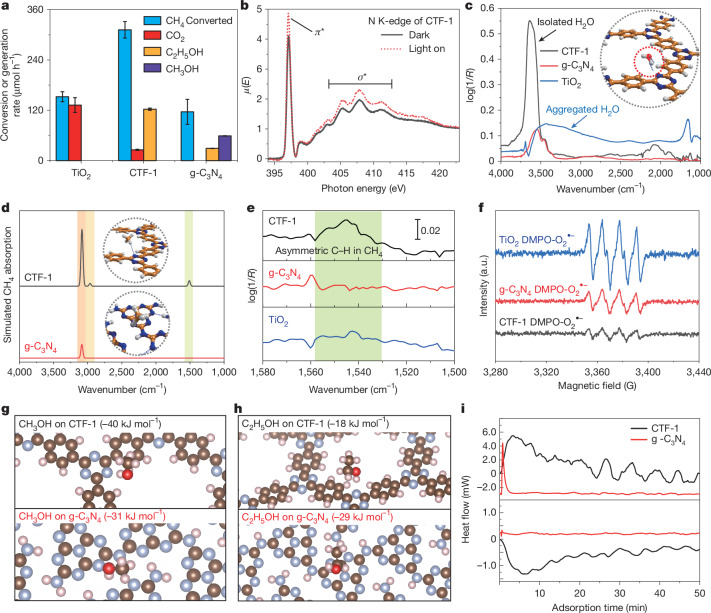
In situ feedstock adsorption and product desorption on CTF-1, g-C_3_N_4_ and TiO_2_. **a**, Comparison of photocatalytic activities of methane transformation on CTF-1, g-C_3_N_4_ and TiO_2_. **b**, N *k*-edge NEXAFS spectra of CTF-1 in the presence or absence of LED light (320 nm) irradiation. **c**, DRIFTS over H_2_O-saturated CTF-1, g-C_3_N_4_ and TiO_2_ at 25 °C. Inset, favourite water adsorption site on CTF-1 modelled by DFT (C, N, H and O atoms are shown in gold, blue, white and red, respectively). **d**, Calculated CH_4_ FTIR signals on CH_4_-saturated CTF-1 (mainly on benzene units) and g-C_3_N_4_ (on triazine units). **e**, Enlarged DRIFTS of CH_4_-saturated CTF-1, g-C_3_N_4_ and TiO_2_ at 25 °C. **f**, In situ 5,5-dimethyl-1-pyrroline-N-oxide (DMPO)-O_2_^•−^ spin-trapping electron spin resonance (ESR) spectra over CTF-1, g-C_3_N_4_ and TiO_2_ in methanol under 90-s LED irradiation (*λ* = 365 nm, 10 W). **g**, Calculated methanol adsorption site and energy on CTF and g-C_3_N_4_. **h**, Calculated ethanol adsorption site and energy on CTF-1 and g-C_3_N_4_ by in silico models. **i**, Calorimetric measurements of methanol and ethanol competitive adsorptions over CTF-1 and g-C_3_N_4_. Adsorption heat flow of methanol on ethanol-saturated catalysts’ surface (top) and ethanol on methanol-saturated catalysts’ surface (bottom). Error bars were obtained by three tests of each sample synthesized from three different batches. Source Data

The distribution of products generated on CTF-1 was then compared with the product distribution obtained with the standard photocatalysts anatase TiO_2_ and g-C_3_N_4_ (Fig. 3a), which both convert less than half of the methane converted with CTF-1. Furthermore, TiO_2_ generates only CO_2_, and g-C_3_N_4_ shows only 20% ethanol yield compared with CTF-1. To clarify the reason for the high conversion and high selectivity towards ethanol over CTF-1, charge transfer as the first step of the overall photocatalytic reaction was investigated by near-edge-X-ray-absorption-fine-structure (NEXAFS) spectroscopy. As indicated by the data in Fig. 3b and Supplementary Fig. 27, when the CTF-1 catalyst was irradiated by LED light, photoelectrons accumulated around the carbon sites of the benzene motifs and photo-holes were left in the nitrogen sites of the triazine units, respectively.

The photoluminescence intensity of CTF-1 is much weaker than that of g-C_3_N_4_ (Supplementary Fig. 28). Considering the similar light absorption capacities of the two materials at 365 nm (Supplementary Fig. 29), the lower photoluminescence intensity of CTF-1 is probably associated with a lower charge recombination rate, because of the better charge separation by the molecular heterojunction of CTF-1, which is consistent with the simulations (Fig. 1a,b) and the higher methane conversion rate. As discussed above, water is a key promoter for methane activation and the strong band seen in the DRIFTS spectra of CTF-1 indicates that it has a high capacity for water adsorption and that most of the adsorbed water molecules are well dispersed rather than aggregated (Fig. 3c). This contrasts with water-saturated TiO_2_ showing a broad peak at around 3,200 cm^−1^ indicative of physisorption of aggregated molecular water, and g-C_3_N_4_ showing the weakest infra-red band indicating weak water adsorption^28,29^ (Fig. 3c). The adsorption sites were also confirmed by ^18^O-labelled water DRIFTS, as noted in Supplementary Fig. 30. As indicated by DFT calculations (inset; Fig. 3c), isolated water molecules are adsorbed through hydrogen-bonded interactions with CTF-1 and two hydrogen bonds are probably formed on CTF-1: one between the N atom of the triazine motif and the H atom of water, and the other between the O atom of water with the H atom of the benzene ring. However, the most favourable adsorption site on g-C_3_N_4_ is the terminal or bridging NH_x_ site due to hydrogen bonding between the O atom of water and the H atom of the NH_x_ species (Supplementary Fig. 31). Thus, the greater extent of water adsorption on CTF-1 than g-C_3_N_4_ has been confirmed by both experiment and simulations, and the unique structure of CTF-1 is crucial for the enhanced water adsorption.

Electron paramagnetic resonance measurements confirm that adsorbed water is readily activated by photogenerated holes to form •OH radicals on the three catalysts. TiO_2_ shows the strongest •OH signal, probably leading to over-oxidation of methane, whereas the relatively weak •OH generation capability of g-C_3_N_4_ indicates its rather low water-activation performance (Supplementary Fig. 32). Compared with TiO_2_ and g-C_3_N_4_, CTF-1 shows an intermediate water-activation potential. Water dissociation $({{\rm{H}}}_{2}{\rm{O}}\to {\rm{H}}{\rm{\bullet }}+{\rm{OH}}{\rm{\bullet }})$ is an endothermic process (Supplementary Fig. 41). DFT calculations show that the energy over the ground-state CTF-1 is +129 kJ mol^−1^, which is 111 kJ mol^−1^ higher than that over g-C_3_N_4_. However, water adsorption is slightly more exothermic on CTF-1 and the barrier for methane activation on CTF-1 is much lower than that on g-C_3_N_4_ (by 136 kJ mol^−1^). Therefore the special activation sites on CTF-1 greatly enhance methane conversion. The in situ DRIFTS difference spectra between water adsorption in the dark and under light irradiation (Supplementary Fig. 33) indicate that the activation of water is more favourable over CTF-1 than over g-C_3_N_4_. It is also noted that water activation over TiO_2_ is the most favourable, but results in CO_2_ as the main (over-oxidized) product. The surface temperature of the catalysts varies between 62 °C and 67 °C under experimental conditions as shown in Supplementary Fig. 34. A DRIFT spectrum over humidified CTF-1 at 65 °C was then measured (Supplementary Fig. 35). The intensity of the water adsorption peak at 65 °C under dark conditions is similar to that at room temperature, indicating that the negative peak under light irradiation in Supplementary Fig. 33 is due to photoexcitation rather than thermal effects from light irradiation. Moreover, no methane oxidation products are observed in the dark at 65 °C, indicating that water activation is driven by photons instead of heating. Therefore, CTF-1 is more efficient than g-C_3_N_4_ for photocatalytic water dissociation, and thus for methane activation under light irradiation.

The simulation results for CH_4_ adsorption (Fig. 3d) indicate different adsorption configurations of methane on the catalyst surface. DRIFTS shows that the CTF-1 sample has a special peak at approximately 1,541 cm^−1^ (Fig. 3e), which is negligible on TiO_2_ and g-C_3_N_4_, consistent with the simulated adsorption configuration. Examination of the analytical frequencies by DFT simulations indicates that a combination of symmetrical and asymmetrical H–C–H bending modes is responsible for this peak, with a distinct simulated band arising around 1,512 cm^−1^ (Fig. 3d), which indicates a relatively strong methane adsorption on CTF-1. Furthermore, as shown in Supplementary Fig. 36 without the dissociated water on the surface of CTF-1, methane exhibits only physical adsorption and its further activation is blocked by a high energy barrier, whereas after water dissociation on the surface of CTF-1, the methane activation barrier is reduced by 186 kJ mol^−1^ (Supplementary Fig. 25). Therefore, the dissociated water greatly facilitates the formation and adsorption of methyl radicals on CTF-1.

The O_2_^•−^-trapping electron paramagnetic resonance indicates that, among the three photocatalysts, CTF-1 shows the weakest signal for the active O_2_ species (Fig. 3f)^30^. The isothermal adsorption and calorimetric measurements (Supplementary Fig. 37) also confirm that CTF-1 adsorbs a much lower amount of O_2_ than TiO_2_. As shown in Fig. 2c, part of the CO_2_ is formed due to the over-oxidation of CH_4_ by O_2_. The low concentration of O_2_^•−^ species thus limits the amount of over-oxidation to CO_2_ on the CTF-1 photocatalyst and leaves instead an opportunity for •CH_3_ coupling towards C_2_ products.

To further rationalize the selectivity of CTF-1 to ethanol over methanol, the binding energies of the two potential species were further assessed by DFT calculations (Fig. 3g,h). The results indicate that the alcohol molecules are stabilized on the benzene motif rather than the triazine motif of CTF-1, in contrast to that in g-C_3_N_4_. Furthermore, the methanol adsorption energy on CTF-1(−40 kJ mol^−1^) is greater than that on g-C_3_N_4_ (−31 kJ mol^−1^), whereas the reverse is true for the ethanol adsorption: far more exothermic on g-C_3_N_4_ (−29 kJ mol^−1^) than on CTF-1 (−18 kJ mol^−1^), which was also confirmed by calorimetric analysis (Fig. 3i). The full details of the calculated energies are given in the Supplementary Table 5). When methanol was introduced on ethanol-saturated CTF-1 (black line in top panel; Fig. 3i) and g-C_3_N_4_ (red line in top panel; Fig. 3i), the overall heat flow represents the heat exchange for methanol adsorption and ethanol desorption. The total energy change is positive (exothermic) during methanol adsorption with simultaneous desorption of ethanol on both catalysts, but the effect is much greater on CTF-1. Whereas ethanol adsorption with methanol desorption is negative (endothermic) only on CTF-1 (black line in bottom panel). Therefore, methanol binds much more strongly than ethanol on CTF-1, whereas the opposite is true on g-C_3_N_4_. The observation indicates that ethanol can be desorbed more readily after generation (to avoid its over-oxidation), while methanol is bound strongly to the surface of the CTF-1 catalyst. This, combined with reactivity differences discussed later, results in the changed selectivity of the released products.

Pulsed chemisorption was then carried out to quantify the irreversible adsorption of methanol and ethanol (Supplementary Figs. 38 and 39). CTF-1 shows a capacity to adsorb 16 times more methanol than g-C_3_N_4_. More importantly, the difference in the capacity of methanol and ethanol adsorption on CTF-1 is 15 times more than that on g-C_3_N_4_. Thus, the relatively high selectivity towards ethanol over CTF-1 is also driven by the sorption properties of the species. After introducing methanol into the feed gas, ethanol generation shows no evident change, but the CO_2_ generation rate increases significantly (Supplementary Fig. 40). Thus, methanol is preferentially over-oxidized to CO_2_ rather than reacting with methyl radicals to form ethanol on the CTF-1 catalyst, which also indicates that there are different activation sites for ethanol generation and methanol over-oxidation. It is believed that CO_2_ produced over CTF-1 most likely results from the over-oxidation of CH_3_OH formed.

DFT was used to calculate reaction profiles for methane to methanol conversion by both CTF-1 and g-C_3_N_4_ (Supplementary Figs. 41 and 42). Calculations indicate that methane activation is the rate-determining step in g-C_3_N_4_ with an activation barrier of 244 kJ mol^−1^, whereas the rate-limiting step for CTF-1 is the formation of the CH_2_OH species (TS_CH2OH_), with a barrier of 190 kJ mol^−1^. This barrier is not present in the g-C_3_N_4_ profile, since a barrierless CH_2_:OH bond formation step follows a concerted hydrogen transfer barrier from the methyl radical to the bonded hydroxyl. On the contrary, CTF-1 presents the competing rate-limiting ethane activation (TS,_C2H5,OH_) and the ethanol formation (TS,_EthOH_) barriers of 149 and 143 kJ mol^−1^, respectively, which are much lower that the rate-limiting step for methanol formation. This explains the observed preference for C_2_ over C_1_ by CTF-1.

**Fig. 4:**
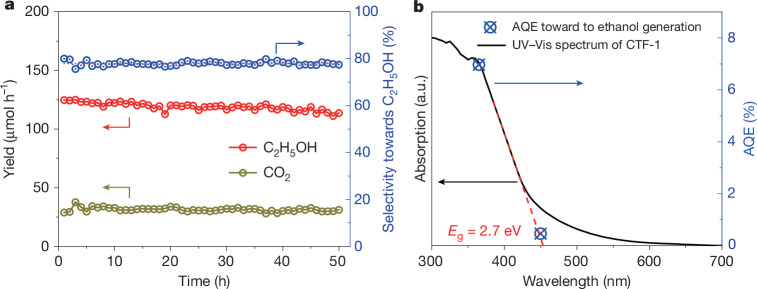
Long-run photocatalytic methane transformation by CTF-1. **a**, Long-term product generation and C_2_H_5_OH selectivity during photocatalytic methane transformation by CTF-1. Reaction conditions, 100-W 365-nm LED irradiation; DGFR = 2,000 ml h^−1^, 16:1 methane (20% methane/argon) to oxygen (water-saturated air, 20% oxygen/nitrogen) flow ratio. **b**, AQE (blue dots) of photocatalytic methane transformation to ethanol by CTF-1 under 365-nm LED and 450-nm LED irradiation. The ultraviolet–visible (UV–Vis) absorption spectrum (black line) of CTF-1 was superimposed for comparison. The bandgap (*E*_g_) of CTF-1 is presented as the intersection of the tangent of the absorption spectrum (red dashed line) with the wavelength axis. Source Data

Finally, the stability of the catalyst was studied (Fig. 4a). Long-term methane conversion used water-saturated 16:1 CH_4_/O_2_ gas mixture with a DGFR of 2,000 ml h^−1^. Under 365 nm LED irradiation, methane is converted continuously over a period of 50 h when the ethanol production rate decreases from 124 μmol h^−1^ to 117 μmol h^−1^ after 30 h reaction, which is probably due to the decayed light intensity caused by a hotter bulb after a long time run, as a 7% decrement in the light intensity of the current light source was detected when the LED was working for 50 h. The 50-h methane conversion reaction results in 12,000 μmol of methane converted. Only two products, ethanol and CO_2_, are observed by GC–FID and the ethanol selectivity remains constant between 75.6% and 80.0%. The overall carbon balance is roughly 91%. CTF-1 modified with PtO_x_ co-catalyst also operates stably over 12 h (Supplementary Fig. 43). The long-term performance tests indicate that the framework and the activity of CTF-1 have been rather stable. Solid-state NMR, FTIR and Raman spectra also indicate a similar chemical and crystalline structure for CTF-1 before and after the long-term run (Supplementary Figs. 44–46). Furthermore, as noted in Supplementary Fig. 47, when ^13^C labelled CH_4_ was used as the reactant, no ^12^CO_2_ was detected, indicating that CO_2_ was not produced from any CTF-1 oxidation products, further indicating the stability of the photocatalyst. The only previous study presenting an AQE for the photocatalytic methane conversion to ethanol reported a value of 0.3% (ref. ^12^), whereas it is 6.9% for CTF-1 at 365 nm and increases to 9.4% when loading a Pt co-catalyst, which is probably an underestimate as multi-electron processes might be involved.

In summary, we have demonstrated that the intrinsic intramolecular heterojunction in the CTF-1 polymer is highly selective for solar-driven methane transformation towards ethanol. At a DGFR of 2,000 ml h^−1^, photocatalytic methane-to-ethanol conversion at 65 °C represents a very high selectivity of around 80% and a yield of 122.4 μmol h^−1^, corresponding to an unprecedented AQE of approximately 6.9% together with the excellent stability. Pt loading on the polymer further improves the AQE to 9.4%. Such activity and selectivity towards ethanol can be ascribed to the intrinsic and simultaneous charge separation by the intramolecular heterojunction, stronger water adsorption than methane, highly selective water-promoted C–H bond cleavage, favourable reaction sites on the benzene motif and the preferred desorption of ethanol to methanol on the optimized CTF-1 catalyst. Overall, these features enable efficient methane transformation to ethanol through an ethane intermediate and avoid the over-accumulation of strong oxidants that probably limit the performance of g-C_3_N_4_-based catalysts. We anticipate that the performances we report here and our mechanistic insights will inspire further exploration of ‘intramolecular heterojunctions’ as the basis of selective and stable photocatalysts for C–C coupling. Although photocatalytic processes could in principle meet imminent industrial demands for net-zero fuels and chemical synthesis, we note that this would require substantial further development and improvements in overall efficiency.

## Methods

### Preparation of CTF-1

The photocatalyst CTF-1 was synthesized by a modified microwave-assisted approach. Briefly, 10 ml trifluoromethanesulfonic acid (Sigma-Aldrich, reagent grade 98%) and 3 g terephthalonitrile (Sigma-Aldrich, 98%) were mixed in a 100-ml polytetrafluoroethylene liner (CEM). The liner was then protected by a sleeve, sealed by CEM standard frame support module and transferred into a microwave oven (MARS 230/60 Microwave Accelerated Reaction System, CEM). The solvothermal reaction was preset to 25 min of ramping time and 60 min of holding at 115 °C. The output power was adjusted automatically to maintain the temperature and ramp rate. After cooling to room temperature, the bulk of the yellow solid was ground into particles. Particles were then washed with acetonitrile at 70 °C three times to remove unreacted precursor and washed with deionized water a few times until neutral to remove the acid solvent. Finally, particles were dried in a vacuum oven in glass vials at 180 °C to remove residual solvent and excess trifluoromethanesulfonic acid overnight.

### Pt depositions

First, 200 mg H_2_PtCl_6_·6H_2_O (Sigma-Aldrich, ACS reagent, at least 37.50% Pt basis) was dissolved in 10 ml deionized water. Then, in each batch, 100 mg CTF-1 was suspended in 160 ml 10 vol% methanol/water in a 450-ml gas-tight glass reactor (Beijing Perfectlight); 400 μl prepared chloroplatinic acid aqueous solution was added into the suspension as the platinum precursor, which contains roughly 3 wt% Pt to CTF-1 or TiO_2_. After a 1-h 300 W Xenon lamp irradiation (Newport), hydrogen was detected by GC equipped with a molecular 5A column and thermal conductivity detector (Varian GC430). The powder colour changed to light grey and was separated by centrifugation. The synthesized powder was washed with deionized water five times and dried in a vacuum oven at 70 °C overnight.

### Ru depositions

First, 200 mg RuCl_3_·xH_2_O (Sigma-Aldrich, ACS reagent, 38–42% Ru basis) was dissolved in 10 ml deionized water. Then, in each batch, 100 mg CTF-1 was suspended in 160 ml 10 vol% methanol/water in a 450-ml gas-tight glass reactor (Beijing Perfectlight); 400 μl prepared RuCl_3_ aqueous solution was added into the suspension as the Ruthenium precursor, which contains roughly 3 wt% Ru to CTF-1. After a 1-h 300 W Xenon lamp irradiation (Newport), the powder colour changed to light grey and was separated by centrifugation. The synthesized powder was washed with deionized water five times and dried in a vacuum oven at 70 °C overnight.

### Control photocatalyst

Anatase TiO_2_ nanopowder with an average particle size of 20 nm was purchased from Millennium. g-C_3_N_4_ was prepared by calcination of urea in a muffle furnace at 550 °C for 4 h and SiO_2_ (325 mesh) was purchased from Sigma-Aldrich.

### Photocatalytic activity tests

The photocatalytic reactions were carried out in a laboratory-built polytetrafluoroethylene reactor with a quartz window, irradiated by a 365-nm LED source (Beijing Perfecting technology, catalogue no. PLS-LED 100, *λ* = 365 nm) with a light intensity of 100 mW cm^−2^. The light intensity was measured using a power meter (Newport, catalogue no. 1918-R) at the location where the catalyst would be irradiated. The distance between the light source and the reactor window was 3 cm. The reactor used was made of polytetrafluoroethylene with a quartz window and the catalyst bed is shown in Supplementary Fig. 48. The exposed irradiation area of the reactor was 3.14 cm^2^. The upper and bottom part of the reactor was fixed by screw threads. Gas tightness was insured by a rubber ring between the upper and bottom parts of the reactor. Photocatalysts (1 g) were packed tightly between the quartz window and the polytetrafluoroethylene body and the exposing area was the same area as the irradiation window. The volume of the flow reactor was 0.6 ml. The gas lines were connected to the reactor using stainless steel tubing (1/8 inch (317.5 mm)) and Swagelok tube fittings; 20% CH_4_/Ar (BOC), water-saturated simulated air (20% O_2_/N_2_, BOC, zero grade no impurities) and argon (BOC, zero grade) were used as feedstocks. The gas flow rates were controlled by Bronkhorst mass flow meters in the range 1–500 standard cubic centimetres per minute, respectively. The total flow rate under the optimized conditions was 40 standard cubic centimetres per minute. The actual flow rates of methane and oxygen were determined by GC in the dark, the values of which are shown in Table 1 and Supplementary Tables 1 and 2. The outlet gases were monitored by an Agilent 7820 gas chromatograph equipped with online injection valves, a thermal conductivity detector for H_2_, O_2_, N_2_, CO, CO_2_ and CH_4_ detection and FID for CH_4_, CH_3_OH and C_2_H_5_OH detection. For online MS, we used a dynamic sampling mass spectrometer system (HPR-20 type quadrupole mass spectrometer; Hiden Analytical) with integrated quartz inlet capillary. Detector type, single filter dual Faraday/electron multiplier; typical detector sensitivity, 100 ppb (subject to spectral interference); typical response time, less than 300 ms; ultra high vacuum 60 l s^−1^ turbomolecular pump set; heated direct source inlet. Before measurement, filament de-gassing and vacuum component cleaning (until the pressure stabilized at 3 × 10^−8^ mbar) were performed. The chamber was then purged with argon to collect the background until a stable initial baseline was achieved. During measurement, the gas flow rate was kept constant at a DGFR of 2,000 ml h^−1^. A 2-m heated capillary was used to make the detecting gases homogeneous.

The AQE calculation is as follows:

$${\rm{AQE}}( \% )=\frac{\alpha \times {\rm{amount}}\;{\rm{of}}\;{\rm{ethanol}}\;{\rm{generated}}}{{\rm{Total}}\;{\rm{incident}}\;{\rm{photons}}\,(N)}\times 100 \% $$

The proposed oxidation half-reaction is as follows:

$$2{{\rm{CH}}}_{4}+2{h}^{+}\mathop{\longrightarrow }\limits^{\,{{\rm{H}}}_{2}{\rm{O}}}{{{\rm{C}}}_{2}{\rm{H}}}_{6}+2{{\rm{H}}}^{+}$$

The proposed reduction half-reaction is as follows:

$${{{\rm{C}}}_{2}{\rm{H}}}_{6}+{{\rm{O}}}_{2}+2{{\rm{e}}}^{-}+2{{\rm{H}}}^{+}\to {{\rm{C}}}_{2}{{\rm{H}}}_{5}{\rm{O}}{\rm{H}}+{{\rm{H}}}_{2}{\rm{O}}$$

Thus, transferred electrons towards ethanol generation (*α*) = 2

$$N=IA\frac{\lambda }{hc}.$$

Here, *I* is light intensity = 100 mW cm^−2^; *A* is irradiation area = 3.14 cm^2^; *λ* is wavelength of the LED light source = 365 nm; *h* is Planck’s constant = 6.63 × 10^−34^ J s; and *c* is speed of light = 3 × 10^8^ m s^−1^. Thus, the AQE for ethanol generation from methane oxidation could be calculated as follows.

$$\Rightarrow {\rm{AQE}}=\frac{{2\times 2{\rm{\mu }}{\rm{mol}}\min }^{-1}\times {10}^{-6}\div60\times 6.02\times {10}^{23}}{100\,{\rm{mW}}\,{{\rm{cm}}}^{-2}\times {10}^{-3}\times 3.14\,{{\rm{cm}}}^{2}\times \frac{365\,{\rm{nm}}\times {10}^{-9}}{6.63\times {10}^{-34}\,{\rm{J}}\,{\rm{s}}\times 3\times {10}^{8}\,\text{m}{{\rm{s}}}^{-1}}}\times 100 \% $$

$$\Rightarrow {\rm{AQE}}(365\,{\rm{nm}})=6.97 \% $$

The isotopic labelling experiment used a batch reactor (Supplementary Fig. 49) to obtain high concentrations of products and to save isotopic reagents. A Shimadzu GC–MS instrument (GCMS-QP2010 SE) was used for the analysis. ^13^C measurements were carried out in a 100 ml quartz reactor with ^13^CH_4_ (Sigma-Aldrich) and simulated air (20% O_2_/N_2_, BOC) on humidified CTF-1 catalysts. In detail, 1 g photocatalyst was first dispersed on the bottom of the reactor. Then, the reactor was purged by water-saturated argon for 30 min. The reactor was then put in an oven at 65 °C for 1 h to simulate the humidified atmosphere in the flow reactor. Then, 12 ml ^13^C labelled methane and 4 ml simulated air were injected into the reactor with a CH_4_:O_2_ ratio of 16:1; 1 ml gas in the reactor was injected into GC–MS to obtain spectra before the reaction. Finally, the reactor was irradiated by the same LED light source as the activity tests for 30 min, and 1 ml of the product gas was injected into the GC–MS to obtain spectra after reaction.

Measurements using ^18^O were carried out in the batch reactor with H_2_^18^O (Sigma-Aldrich, 99%). After 1 g photocatalyst was dispersed on the bottom of the reactor, the reactor was purged with a mixed gas of CH_4_ (20% CH_4_/Ar, BOC) and simulated air (20% O_2_/N_2_, BOC) at CH_4_:O_2_ = 16:1 for 30 min. The reactor was then placed into an oven at 65 °C for an hour. After that, 2 μl H_2_^18^O was injected by a 5-μl Hamilton syringe into the reactor. The reactor was placed into the oven at 65 °C for another 1 h to confirm the homogeneous adsorption of water on the catalyst surface. Then, 1 ml of gas in the reactor was injected into GC–MS to obtain the spectra before the reaction. Finally, the reactor was irradiated by the same LED light source as activity tests for 30 min, and the product gas was injected into GC–MS to obtain spectra after the reaction.

### Catalyst characterizations

Powder X-ray diffraction measurements were made by a SAXSLAB Ganesha 300XL small-angle X-ray scattering system in wide angle X-ray scattering mode with a range from 2*θ* = 2°–40° (wavelength 0.154 nm, Cu Kα radiation). Attenuated total reflection FTIR spectroscopy was collected by a Shimadzu IRAffinity-1s spectrometer with a Specac Quest (Germanium) attenuated total reflection accessory at a range of 400–4,000 cm^−1^. ^13^C cross-polarization magic-angle spinning solid-state NMR spectra were collected at ambient temperature on a BRUKER Advance 300 WB spectrometer (Bruker UK Ltd) with a 4-mm magic-angle spinning probe. Solution NMR spectra were measured using a Bruker Avance Neo (700 MHz) and ^1^H NMR spectra were referenced to residual protiated solvent at *δ* 7.26 (CDCl_3_). X-ray photoelectron spectroscopy (XPS) was conducted on a Thermo Scientific XPS K-alpha machine using monochromatic Al Kα radiation. Survey scans were collected in the range of 0–1,100 eV (binding energy) at a pass energy of 160 eV. High-resolution scans were recorded for the main core lines at a pass energy of 20 eV. Scans were analysed using CasaXPS software. Raman spectra were measured on a Renishaw InVia Raman Microscope using a 325-nm excitation laser, between 100 and 3,500 cm^−1^. UV–Vis absorption spectra were obtained on an Agilent Carry 3500 UV–Vis–near infra-red spectrophotometer fitted with an integrating sphere. Reflectance measurements were performed on powdered samples, using a standard barium sulfate powder as a reference. The reflection measurements were converted to absorption spectra using the Kubelka–Mulk transformation. Thermogravimetric analyses were carried out under ambient conditions (25 °C, 1 bar) with Setsys from Setaram Instrument to study the reactant adsorption properties of the catalysts. In situ ESR signals of radicals trapped by DMPO were obtained using an MS-5000 Magnettech ESR spectrometer. The spectra were taken from 20 µl methanol solution containing 20 mM DMPO, with a catalyst concentration of 5 mg ml^−1^, under 90 s of LED irradiation (*λ* = 365 nm; 10 W). Measurement parameters were as follows: centerfield, 3,375 G; sweep width, 200 G; microwave frequency, 9.74 GHz; microwave power, 20 mW. DRIFTS experiments without or with 100-W high-pressure Hg arc lamp (Oriel 6281) irradiation were carried out using a Thermo Scientific Nicolet iS50 FTIR spectrometer with a mercury cadmium telluride detector at a scan number of 128 and a resolution of 4 cm^−1^. The spectrometer was equipped with a Harrik Praying Mantis diffuse reflection accessory and a Harrick high-temperature reaction chamber with ZnSe windows. The reaction cell was connected to an SH-110 dry scroll vacuum pump (Agilent Technologies). H_2_O in a quartz tube welded with Kovar was purified by repeated cycles of freeze–pump–thaw treatments before use. Typically, 50 mg catalyst was loaded in the sample holder of the reaction cell, heated in Ar at 200 °C for 1 h, cooled to room temperature and evacuated, and the spectrum was recorded as the background spectrum. Desired gases were then admitted to reach steady-state adsorption and the DRIFTS spectra were measured. Calorimetric measurements of methanol and ethanol competitive adsorptions were carried out using a Setaram Sensys EVO 600 DSC microcalorimeter. Catalysts (50 mg) in the sample quartz tube was degassed at 200 °C for 1 h in an Ar flow of 30 ml min^−1^ and cooled to 298 K. The flow was first switched to an Ar flow bubbled through a saturator filled with liquid methanol (ethanol) at 298 K and, after the heat flow became stable, was then switched to an Ar flow bubbled through a saturator filled with liquid ethanol (methanol) at 298 K. Control experiments using an empty quartz tube showed negligible heat flows for both methanol and ethanol. Adsorption microcalorimetric O_2_ isothermal adsorption was measured using a combination of Setaram Sensys EVO 600 microcalorimetry and a Micromeritics Autochem II 2920 chemisorption apparatus. Typically, 50 mg of catalysts in the sample quartz tube was degassed at 200 °C for 1 h under He flow of 50 ml min^−1^ and cooled to −100 °C. The flow was then switched to 5% O_2_/He at a flow rate of 50 ml min^−1^ for O_2_ adsorption. CH_3_OH and CH_3_CH_2_OH pulse adsorptions were measured on a chemisorption apparatus (Micromeritics Autochem II 2920) equipped with a vapour generator. A certain amount of catalysts with a bed thickness of 2 mm in the sample quartz tube was pre-treated in the He flow at 100 °C for 60 min and then cooled to 50 °C for 20 min in the ultra-pure He flow before measurement. NEXAFS spectra, with or without 320 nm LED light irradiation in the total electron yield mode, were measured at the Photoemission Endstation (BL10B) in the National Synchrotron Radiation Laboratory (NSRL) in Hefei, China. C K-edge and N K-edge NEXAFS spectra were collected at energies from 275 eV to 300 eV and from 390 eV to 430 eV with a 0.2 eV energy step, respectively. The NEXAFS raw data were processed as follows: first, the photon energy was calibrated from the 4f spectral peak of a freshly sputtered gold wafer, then a line was subtracted to set the pre-edge as zero and finally the spectrum was normalized to yield an edge-jump to one. In situ synchrotron radiation photoionization MS was measured at the combustion beamline (BL03U) of the NSRL. The species in the photocatalytic reactor (2 Torr) were introduced into the ionization chamber in situ (0.01 Pa), crossed and ionized by the synchrotron radiation light at 12 eV or 14.2 eV. The ions generated were sampled into the time-of-flight–MS chamber (1.5 × 10^−5^ Pa) by a set of einzel lens. The ion signals were amplified with a pre-amplifier (VT120C, ORTEC) and recorded using a P7888 multiscaler (FAST Comtec). Synchrotron radiation from the undulator beamline was monochromatized with a 200 lines per millimetre laminar grating (Horiba Jobin Yvon), which covered the photon energy from 7.5 eV to 22 eV with an energy resolving power of 3,000 (*E*/∆*E* at 10 eV). The average photon flux could reach the magnitude of 1,013 photons per second after suppressing the higher-order harmonic radiation by a gas filter filled with noble gas.

### Computational methods

DFT calculations were performed using Perdew–Burke–Ernzerhof functional^15^, as implemented in the Vienna ab initio simulation code based^16–18^ on models containing either four Tris–triazine rings of g-C_3_N_4_ or three alternating triazine and benzene rings of CTF-1 (Supplementary Figs. 1 and 2). Periodic DFT was used to assess the relative adsorption energies of various intermediates in the pathways for CH_4_ conversion to either methanol or ethanol. Models of CTF-1 were compared with models of g-C_3_N_4_ as both materials were shown to be active in the formation of alcohol products, whereas only the CTF-1 was selective to the C_2_ product. The model used for g-C_3_N_4_ consisted of four linear Tris–triazine rings arranged in two chains; the model used for CTF-1 comprised three alternating triazine and benzene rings. The adsorption energies (EA) were calculated according to the following equation:

$${\rm{EA}}=\,{E}_{{\rm{complex}}}-{E}_{{\rm{ads}}}-{E}_{{\rm{surf}}}$$

Where the adsorption energy was determined by subtracting the energies of the neutral adsorbate(s) in a vacuum (*E*_ads_) and the energy of the pristine surface (*E*_surf_) of either CTF-1 or g-C_3_N_4_ from the total energy of the adsorbed complex (*E*_complex_). The resulting values would determine the desorption enthalpies of the neutral species and allow for the assessment of whether the selectivity was a desorption-driven phenomenon. Although several binding sites were considered for each adsorption process only the most energetically favourable are discussed here.

All computational values were derived with the Perdew–Burke–Ernzerhof functional^8^, as implemented using the Vienna ab initio simulation code^13,16,17^. This methodology was applied previously to explain the bulk properties of g-C_3_N_4_, with the models produced for that study forming a basis for the carbon nitride component of this current work^31^. Plane-wave basis sets were applied to the valence electrons of each element with core electrons described by the projected augmented wave method^32^. Long-range non-bonding interactions were assessed by the Grimme D3 empirical dispersion method^33,34^. A fine Monkhorst–Pack grid with *k*-point 5 × 5 × 1 was used to calculate surface wavefunctions, and 15 Å of vacuum was added in the *z *direction for both photocatalyst models. The electronic threshold for the convergence of the self-consistency cycles was set to 10^−5^ eV, with the convergence determined by the Blöchl smearing method^34^. No constraints were set in any of the systems reported here, with the ionic relaxation threshold of 0.01 eV Å^−1^ and a plane-wave cut-off of 520 eV being applied in all cases. Transition state structures were located with aid of the climbing image nudged elastic band approach, whereas intermediate(s) were optimized without constraints in any degree of freedom.

## Online content

Any methods, additional references, Nature Portfolio reporting summaries, source data, extended data, supplementary information, acknowledgements, peer review information; details of author contributions and competing interests; and statements of data and code availability are available at 10.1038/s41586-025-08630-x.

## Supplementary information

Supplementary Information Supplementary characterizations of the materials, DFT calculations, catalyst performances, reactants adsorption results, in situ DRIFTS spectra, synchrotron radiation photoionization MS spectra, other mechanisms investigation results and details of the light source used, and Supplementary Tables 1–5, Figs. 1–49 and references.

## Source data

Source Data Fig. 1

Source Data Fig. 2

Source Data Fig. 3

Source Data Fig. 4

## Data Availability

All data in the Supplementary Information are available from the authors. Source data are provided with this paper.
