# Author Correction: Methane oxidation to ethanol by a molecular junction photocatalyst

**DOI:** 10.1038/s41586-025-09193-7

**Published:** 2025-06-03

**Authors:** Jijia Xie, Cong Fu, Matthew G. Quesne, Jian Guo, Chao Wang, Lunqiao Xiong, Christopher D. Windle, Srinivas Gadipelli, Zheng Xiao Guo, Weixin Huang, C. Richard A. Catlow, Junwang Tang

**Affiliations:** 1 Department of Chemical Engineering, University College London, London, UK; 2 State Key Laboratory of Precision and Intelligent Chemistry, iChEM, Key Laboratory of Surface and Interface Chemistry and Energy Catalysis of Anhui Higher Education Institutes, Department of Chemical Physics, University of Science and Technology of China, Hefei, China; 3 School of Chemistry, University of Cardiff, Cardiff, UK; 4 School of Chemistry, University of Leeds, Leeds, UK; 5 Department of Chemistry, University College London, London, UK; 6 Industrial Catalysis Center, Department of Chemical Engineering, Tsinghua University, Beijing, China; 7 Department of Chemistry, The University of Hong Kong, Hong Kong SAR, China

**Keywords:** Photocatalysis, Natural gas, Catalytic mechanisms

Correction to: *Nature* 10.1038/s41586-025-08630-x Published online 20 January 2025

In the version of this article initially published, the term GHSV (gas hourly space velocity) appeared throughout the text rather than DGFR (dry gas flow rate), as now appears throughout the text and Supplementary information. Supplementary Table 1 has been clarified to show a rounded CH_4_ to O_2_ ratio, and Supplementary Table 2 now includes reactant flow rates and specifies the digital-controlled mass flow meters used in data collection. The changes have been made in the Supplementary information and HTML and PDF versions of the article.

