# Supplementary material for: Methane oxidation to ethanol by a molecular junction photocatalyst
Source: Nature. 2025 Jan 20;639(8054):368–74. doi: 10.1038/s41586-025-08630-x (PMC11903337; doi:10.1038/s41586-025-08630-x)
Supplement: Supplementary file 1 — Supplementary characterizations of the materials, DFT calculations, catalyst performances, reactants adsorption results, in situ DRIFTS spectra, synchrotron radiation photoionization MS spectra, other mechanisms investigation results and details of the light source used, and Supplementary Tables 1–5, Figs. 1–49 and references. [file 41586_2025_8630_MOESM1_ESM.pdf]

nature portfolio

https://doi.org/10.1038/s41586-025-08630-x

Supplementary information

Methane oxidation to ethanol by a molecular junction photocatalyst

In the format provided by the authors and unedited

Supplementary information

Methane oxidation to ethanol by a molecular junction photocatalyst

Jijia Xie,[1] Cong Fu,[2] Matthew G. Quesne,[3,4] Jian Guo,[5] Chao Wang,[1] Lunqiao Xiong, [1,6] Christopher D. Windle,[1] Srinivas Gadipelli,[5] Zheng Xiao Guo,[5,7*] Weixin Huang,[2*] C. Richard A. Catlow,[3,5*] Junwang Tang[1,6*]

[1]Department of Chemical Engineering, University College London, London WC1E 7JE, UK.
[2]Key Laboratory of Precision and Intelligent Chemistry, iChEM, Key Laboratory of Surface and Interface Chemistry and Energy Catalysis of Anhui Higher Education Institutes, Department of Chemical Physics, University of Science and Technology of China, Hefei, 230026, Anhui, China.
[3]School of Chemistry, University of Cardiff, Park Place, Cardiff, CF10 3AT UK.
[4]School of Chemistry, University of Leeds, Leeds LS2 9JT, UK
[5]Department of Chemistry, University College London, London WC1H 0AJ, UK.
[6]Industrial Catalysis Center, Department of Chemical Engineering, Tsinghua University, Beijing 100084, China.
[7]Department of Chemistry and Zhejiang Institute of Research and Innovation (ZIRI), The University of Hong Kong, Hong Kong SAR, China.

*Correspondence to: jwtang@tsinghua.edu.cn (J.T.); catlowr@cardiff.ac.uk or c.r.a.catlow@ucl.ac.uk (C.R.A.C.); zxguo@hku.hk (Z.X.G.); huangwx@ustc.edu.cn (W.H.)

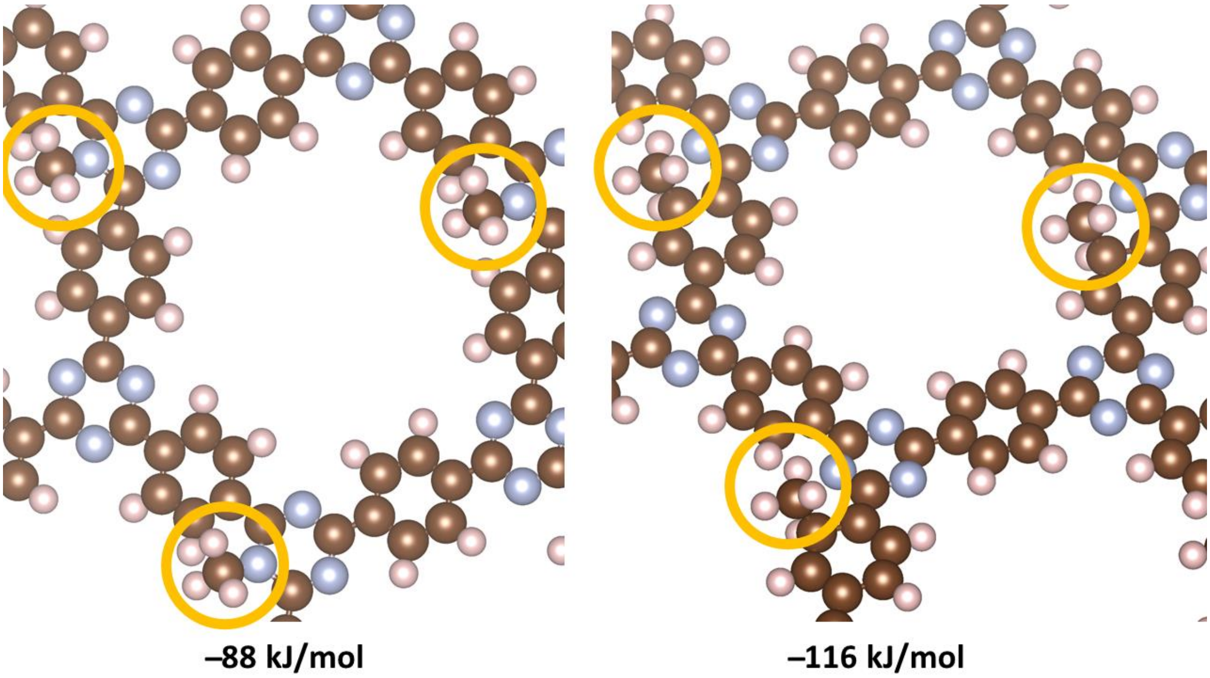

Fig. S1. Two lowest energy adsorption modes for methyl radical on CTF-1.
Supplementary Note to Fig. S1.
The lowest energy adsorption mode (right) is positioned on the benzene ring whilst the second lowest energy mode is positioned on the nitrogen in the triazine ring (left). Energies are given in kJ/mol in relation to the pristine CFT-1 surface and the energy of methane minus half the energy of a hydrogen molecule in the gas phase. It can be noted that the binding to the most favourable site of the benzene motif is 28 kJ/mol more exothermic than to the triazine motif site in CTF-1. The blue, gold and gray denote N, C and H, respectively.

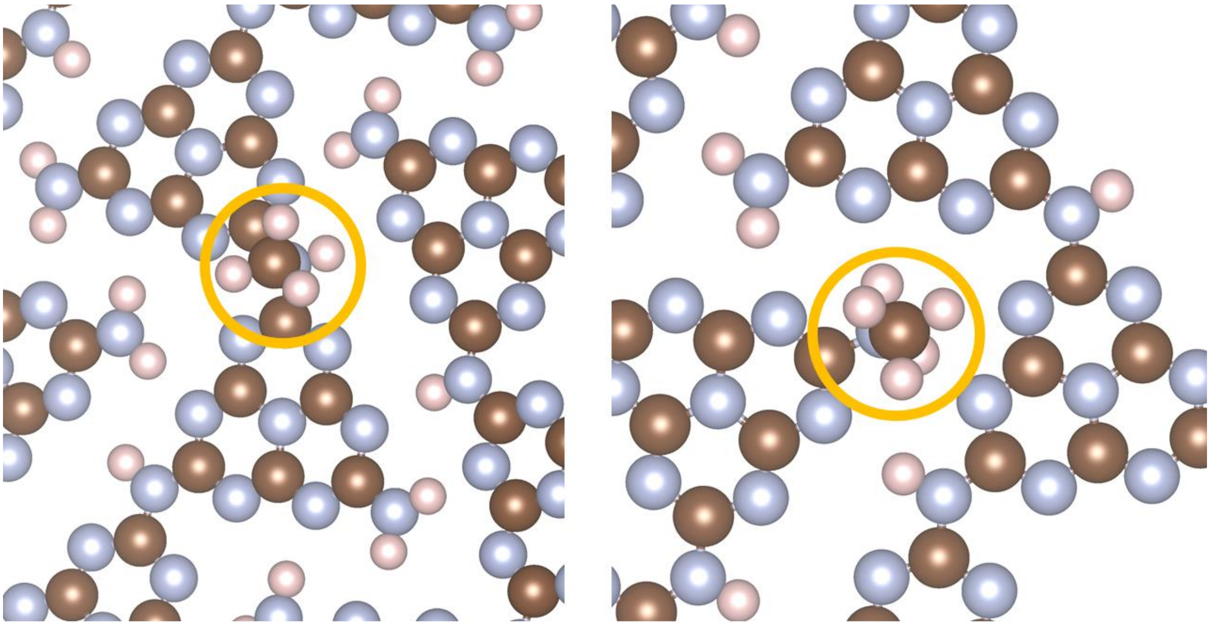

Fig. S2. Two lowest energy adsorption modes for methyl radical on g-C_3N_4.

Supplementary Note to Fig. S2.

The lowest energy adsorption mode (right) is positioned on a terminal NH_2 group whilst the second lowest energy mode is positioned on the linker NH group (left). Energies are given in kJ/mol in relation to the pristine g-C_3N_4 surface and the energy of methane minus half the energy of a hydrogen molecule in the gas phase.

The results from Figs S1 and S2 indicate that the binding of methyl radicals to the most favourable site of the benzene motif of CTF-1 is 55 kJ/mol stronger than the favourable adsorption site in g-C_3N_4.

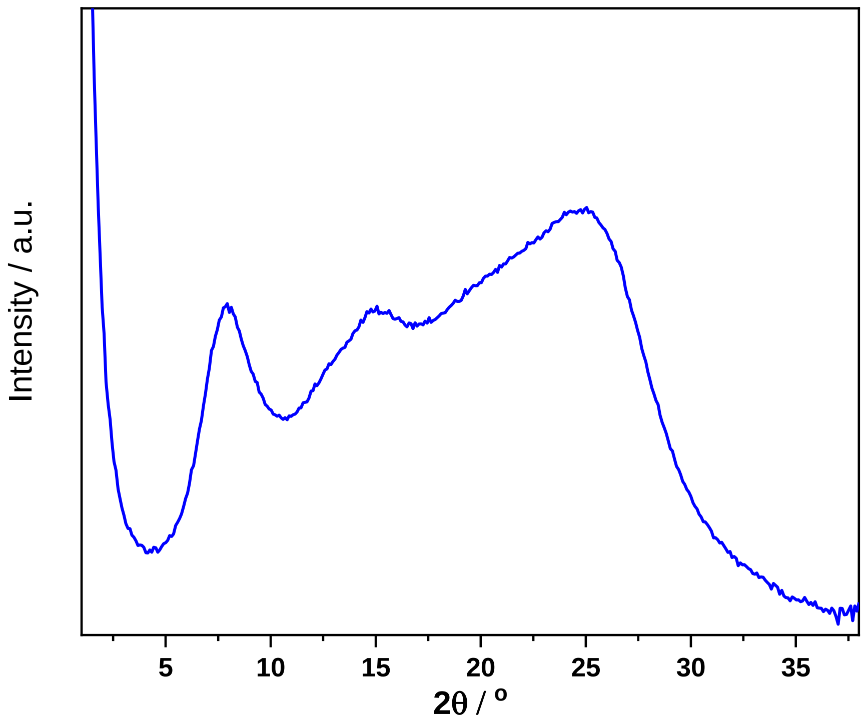

Fig. S3. PXRD pattern for CTF-1 in vacuum.

Supplementary Note to Fig. S3.

The signal drop before 3º is the response of the silica capillary which is the holder of powder samples. The first peak located at *ca.* 7.9º is associated with the hexagonal cages. The peak at *ca.* 24.8 º indicates a multi-layer structure with an interplanar stacking distance of 3.5 Å tested experimentally.[1,2]

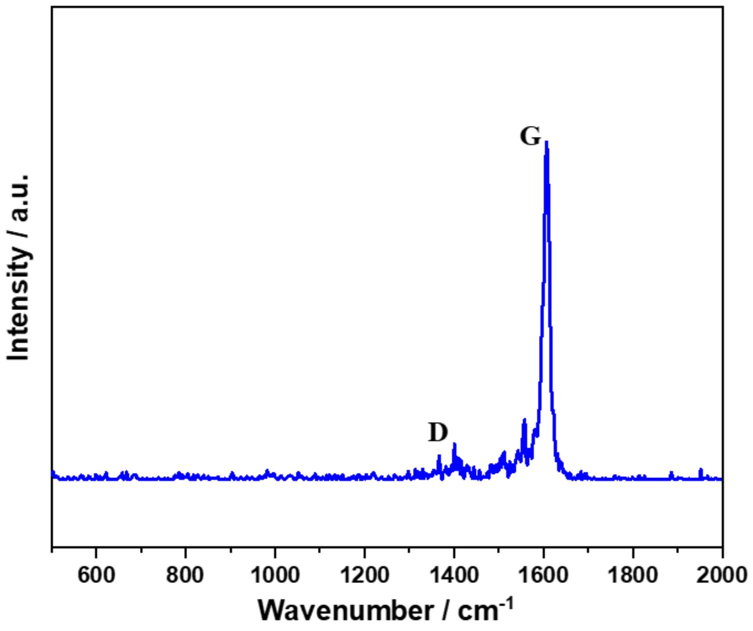

Fig. S4. Raman spectra of CTF-1 excited by 325 nm laser in the atmosphere.

Supplementary Note to Fig. S4.

The Raman spectrum shows a high degree of conjugation of the synthesised photocatalyst, indicating a well-ordered planar structure in each layer. The sharp peak located at *ca.* 1609 cm^{-1} is assigned as G peak, which involves sp^2 carbon in the polymer materials and likely indicates a high degree of conjugation. A small peak at *ca.* 1400 cm^{-1} is assigned as D peak and related to the sp^3 carbon in the structure.[3]

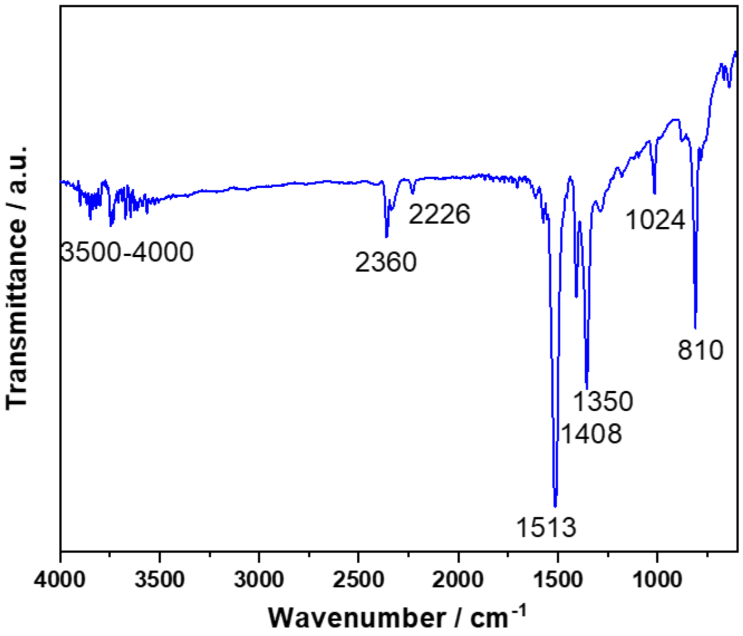

Fig. S5. Experimental ATR-FTIR spectra of CTF-1 in atmosphere.

Supplementary Note to Fig. S5.

The two strongest peaks at 1513 and 1350 cm^{-1} represent C-N bonds stretching mode and the in-plane rings stretching vibrations, respectively, indicating the successful synthesis of triazine rings.[4] A major peak at 1024 cm^{-1} and two small peaks at 1160 and 1272 cm^{-1} are related to C-N bond bending vibrations, confirming the successful polymerisation process.[5] Peaks located at 810, 1408 and 1623 cm^{-1} are associated with benzene rings and can be assigned as the bending vibrations of the out-of-plane C-H bonds, the C-H stretching vibrations and the C-C bonds stretching, respectively.[3] The small peak at 2226 cm^{-1} is resulted by the stretching of the terminal -C≡N groups.[6] Additional peaks at *ca.* 2360 and 3500-4000 cm^{-1} are associated with the surface-adsorbed CO_2 and water in the atmosphere.

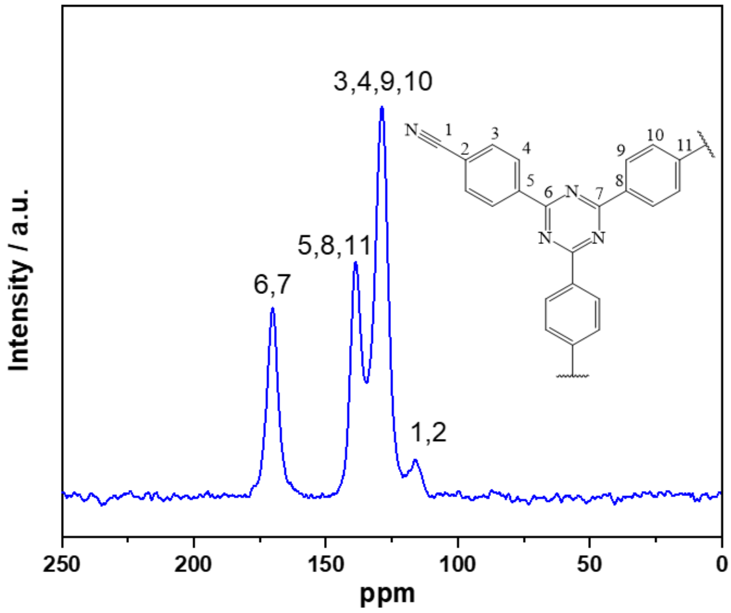

Fig. S6. ^{13}**C ssNMR spectra of CTF-1.**

Supplementary Note to Fig. S6.

The Peaks assigned as 6 and 7 at *ca.* 170 ppm are related to the carbon atoms in the triazine unit. The peak at 139 ppm labelled as 5, 8 and 11 is associated with α-carbon atoms which were connected to the triazine rings. The strongest peak at 129 ppm assigned as 3, 4, 9 and 10 represents carbon atoms in the aromatic ring. The weakest peak at the highest field labelled as 1 and 2 is characteristic of the two types of carbon atoms in the terminal cyanide groups.[7]

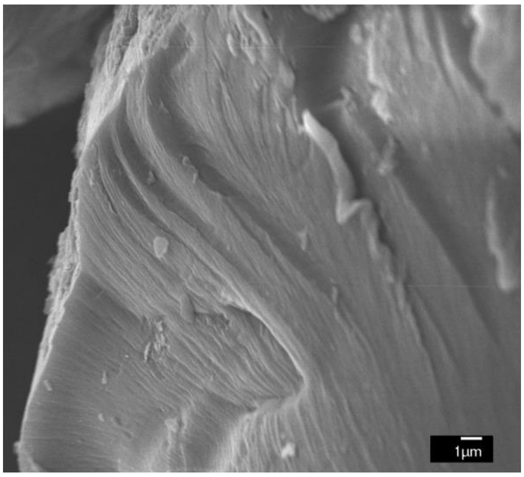

Fig. S7 SEM image of CTF-1.

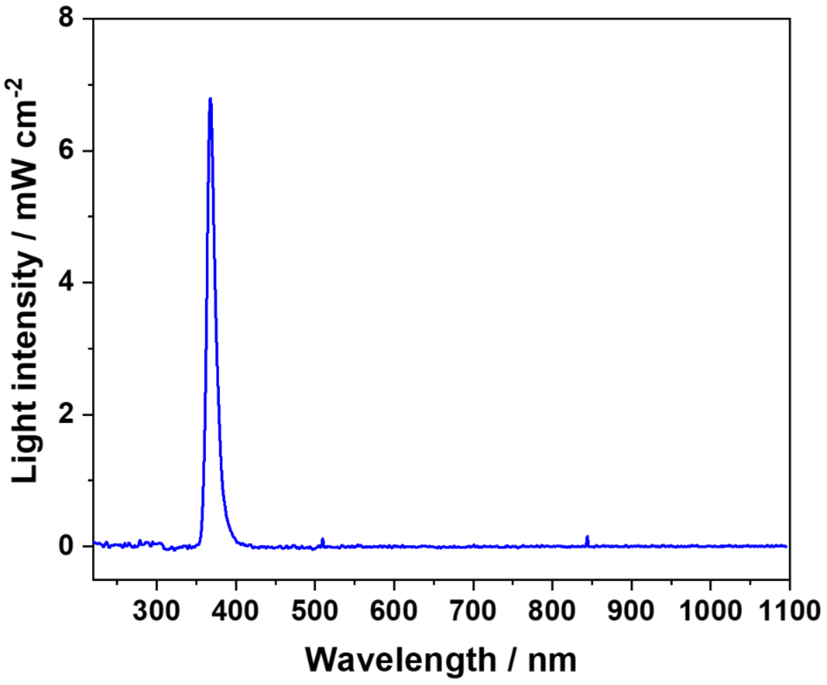

Fig. S8. Spectrum of the light source (Perfectlight, PLS-LED 100, λ = 365nm) under identical experimental conditions.

Supplementary Note to Fig. S8.

The light intensity is calibrated by a Newport power meter (1908-R) under identical experimental conditions.

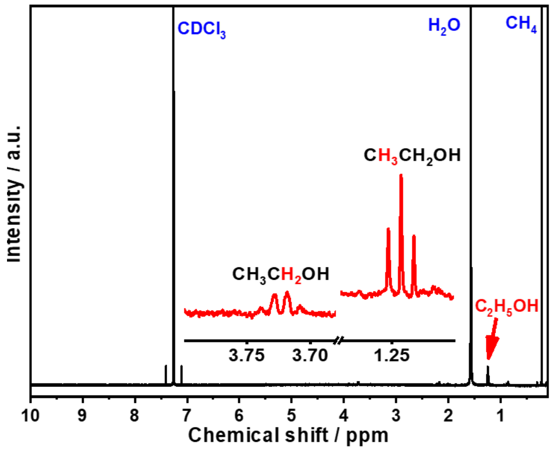

Fig. S9. The ^{1}H NMR spectra (700 MHz) of the outlet gas dissolved in $CDCl_3$.

Supplementary Note to Fig. S9.

Reaction conditions: gas flow rate 40 mL min^{-1}, room temperature and 365 nm LED irradiation. The three strongest peaks located at 0.21, 1.56 and 7.25 ppm are associated with dissolved methane, water and deuterated chloroform, respectively. Two multiplets are generated compared with dark conditions. The triplet at 1.24 ppm is assigned to the CH_3 group bound to a CH_2 group. The quartet at 3.72 ppm are assigned to the CH_2 group bound to α-CH_3 group. Integration of the peaks gives *ca.* a ratio of 3:2 (CH_3/CH_2), indicating the molar ratio of CH_3:CH_2 is 1:1. All these prove that the generated organic product is ethanol.[8]

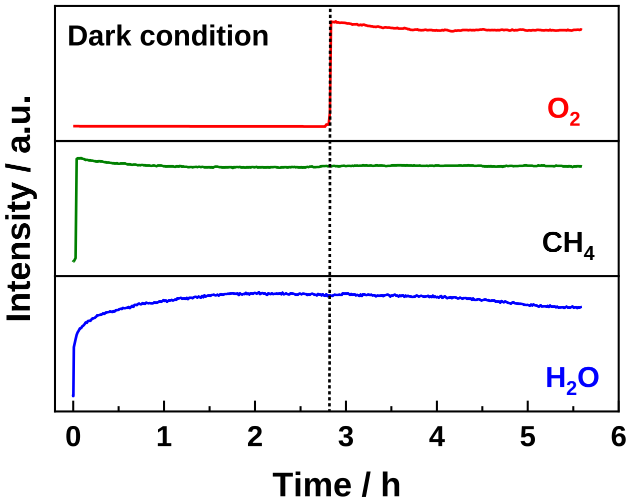

Fig. S10 Mass spectral responses of water (blue line, *m/z* = 18), methane (green line, *m/z* = 16), and oxygen (red line, *m/z* = 32) in the outlet gas under dark conditions over CTF-1. Reaction conditions: gas flow rate 40 mL min^{-1} and 65 ^{o}C.

Supplementary Note to Fig. S10.

The inline MS spectra were recoreded to monitor the change of reactants under dark conditions at 65 ^{o}C. When humidified methane was introduced to the CTF-1 catalyst, the adsorption of methane and water reaches equilibrium in about 3 hours. Subsequently, O_2 gas was introduced into the gas mixture and the overall flow rate was kept at 40 mL min^{-1}. The water signal remains constant in the first hour and then slightly decreases. After 5 hours, the water content reaches equilibrium again. This phenomenon is very similar to that during the photocatalytic process when CH_4, O_2 and H_2O were co-fed into the reactor (Fig. 1E). Hence, the slight decrease of water is unrelated to the partial oxidation of methane, and likely due to the extra adsorption of water by the very porous catalyst.

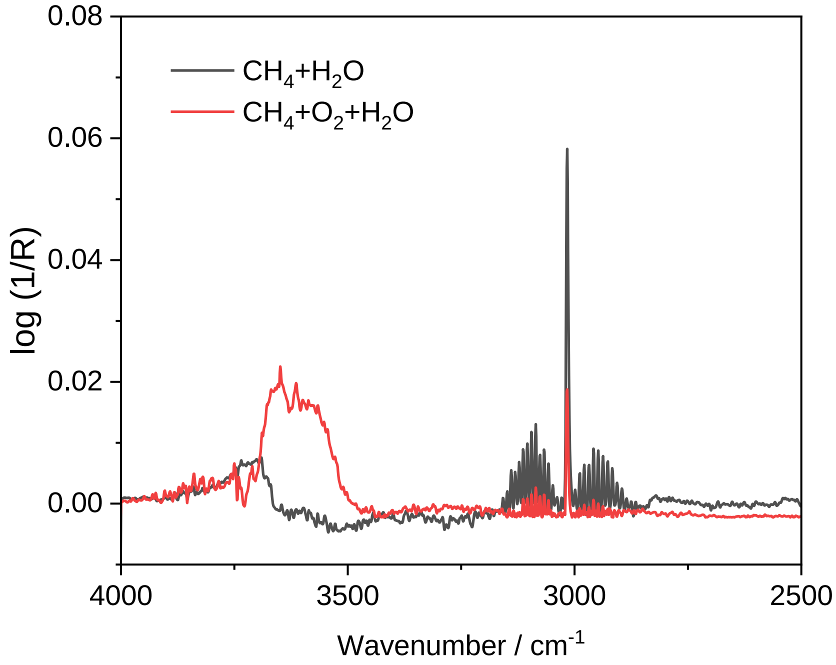

Fig. S11 *In situ* DRIFTS spectra of CTF-1 for the co-adsorption of CH_4+ H_2O and CH_4 + O_2+ H_2O.

Supplementary Note to Fig. S11.

Water adsorption under conditions with and without oxygen gas was investigated by *in situ* DRIFTS. The peaks located at ca. 3015 cm^{-1} represent the methane signal and the peaks located at 3500-3800 cm^{-1} are assigned to adsorbed water. In the presence of oxygen gas, the water signal greatly increases, and new peaks appear at lower wavenumbers besides the previous one, which indicates that new adsorption sites of water molecules are trigged on the surface of CTF-1 in the presence of oxygen.

Table S1. Photocatalytic activity. Comparison of photocatalytic activities of methane transformation on CTF-1 in a packed bed flow reactor under 365 nm LED light irradiation under different reaction conditions.

Entry	Photo-catalyst	CH_4:O_2 [a]	Methane conversion[b] / %	Product selectivity based on carbon[c]/ %			Products yield umol/h			Reactant flow rate mmol/h	
				C_2H_5OH	CH_3OH	CO_2	C_2H_5OH	CH_3OH	CO_2	CH_4	O_2
1	CTF-1	1:1 (1.1:1)	6.1 (±0.2)	3.4 (±0.02)	-	91.7 (±6.6)	10.9 (±0.37)	-	582.0 (±45.8)	10.4 (±0.2)	9.8 (±0.2)
2	CTF-1	4:1 (4.3:1)	3.1 (±0.1)	37.3 (±0.1)	-	44.2 (±1.7)	94.7 (±3.7)	-	218.5 (±10.7)	16.2 (±0.2)	3.8 (±0.2)
3	CTF-1	16:1 (15.8:1)	1.7 (±0.1)	78.6 (±1.7)	-	8.2 (±0.5)	122.4 (±2.6)	-	26.0 (±1.2)	18.9 (±0.5)	1.2 (±0.1)
4	CTF-1	32:1 (32.3:1)	0.7 (±0.1)	78.0 (±1.1)	-	16.3 (±1.2)	50.0 (±5.8)	-	20.9 (±2.8)	19.4 (±0.4)	0.6 (±0.1)
5	SiO_2	16:1	0	-	-	-	-	-	-	-	-
6	CTF-1	100% Air (no CH_4)	-	0	0	0	-	-	-	-	-
7	3 wt.% PtO_x/CTF-1	16:1 (15.7:1)	2.3 (±0.1)	79.6 (±7.0)	-	11.8 (±1.2)	167.6 (±14.7)	-	52.3 (±5.8)	17.3 (±0.3)	1.1 (±0.1)

[a]CH_4 source is 20% CH_4/Ar and O_2 source is air (20% O_2/N_2).
The ratio is the rounded value (either up or down) of the precise ratio presented in parenthesis.
[b]The value is the average methane conversion rate during 4 h light irradiation. The error in the parenthesis is the calculated standard deviation.
[c]The value is the average product selectivity during 4 h light irradiation. The error in the parenthesis is the calculated standard deviation over 3 hours (9 measurements).

Supplementary Note to Table S1.

The methane conversion rate decreases as the concentration of oxygen is reduced while the selectivity towards ethanol is enhanced (Entries 1-4). The highest methane conversion rate is 6.1%, generated continuously at a ratio of CH_4:O_2 =1:1, but the major product is undesirable carbon dioxide. When the CH_4:O_2 ratio increases to 4:1, the ethanol selectivity increases to *ca.* 40%. Further increasing the methane concentration to CH_4:O_2 =16:1 leads to the highest ethanol selectivity

of 78.6% with a methane conversion rate of 1.7%. Continuously increasing the CH_4:O_2 ratio to 32:1 results in no evident change in the ethanol selectivity, but the methane conversion rate is more than halved compared with the 16:1 (CH_4:O_2) entry, which is probably due to the reduced mass transfer of oxygen to the catalyst surface. Thus, 16:1 is the optimised ratio for the fluid system. Two control experiments were also conducted, as shown in Entries 5 and 6; there is no methane transformation or ethanol generation detected in the absence of either a light harvesting material or methane. These data show that the final product, ethanol, is from methane, driven by photocatalysis on CTF-1.

Table S2 Photocatalytic activity. Comparison of photocatalytic activity of methane transformation on CTF-1 in a packed bed flow reactor under 365 nm LED light irradiation under different flow rates.[a]

Entry	Photo-catalyst	DGFR (EFR) / mL h^{-1}	Methane conversion[b] / %	Product selectivity based on carbon[c]/ %			Products yield umol/h			Reactant flow rate mmol/h		
				C_2H_5OH	CH_3OH	CO_2	C_2H_5OH	CH_3OH	CO_2	CH_4	O_2	CH_4/O_2
1	CTF-1	1000 (1066)	2.5 (±0.3)	60.4 (±4.0)	-	35.1 (±6.9)	63.4 (±8.4)	-	35.1 (±6.9)	9.4 (±0.3)	0.59 (±0.06)	15.9
2	CTF-1	2000 (2143)	1.7 (±0.1)	78.6 (±1.7)	-	8.2 (±0.5)	122.4 (±2.6)	-	26.0 (±1.2)	18.9 (±0.5)	1.18 (±0.11)	16.0
3	CTF-1	6000 (6440)	0.3 (±0.02)	66.9 (±7.5)	-	13.0 (±5.4)	47.7 (±7.1)	-	16.4 (±7.5)	56.8 (±1.4)	3.52 (±0.25)	16.1
4	CTF-1	10000 (11951)	0.1 (±0.01)	60.9 (±28.6)	-	10.9 (±4.2)	22.8 (±14.6)	-	9.5 (±3.4)	105.4 (±3.6)	6.51 (±0.35)	16.2

[a]CH_4 source is 20% CH_4/Ar and O_2 source is air (20% O_2/N_2).
[b]The value is the average methane conversion rate during 4 h light irradiation. The error in the parenthesis is the calculated standard deviation.
[c]The value is the average product selectivity during 4 h light irradiation. The error in the parenthesis is the calculated standard deviation over 3 hours (9 measurements).

Supplementary Note to Table S2.
The total inlet dry gas flow rate (DGFR) was set by digital-controlled mass flow meters.
The flow rate in the parenthesis was estimated by the reactant flow rates (EFR) measured at the outlet of the reactor by a soap film flow meter with water vapor included.
The effects of the flow rate at this ratio of reagents were investigated at different DGFR from 1000 to 10000 mL h^{-1} as shown in entries 1-4. The highest methane conversion rate is achieved with a DGFR of 2000 mL h^{-1}.

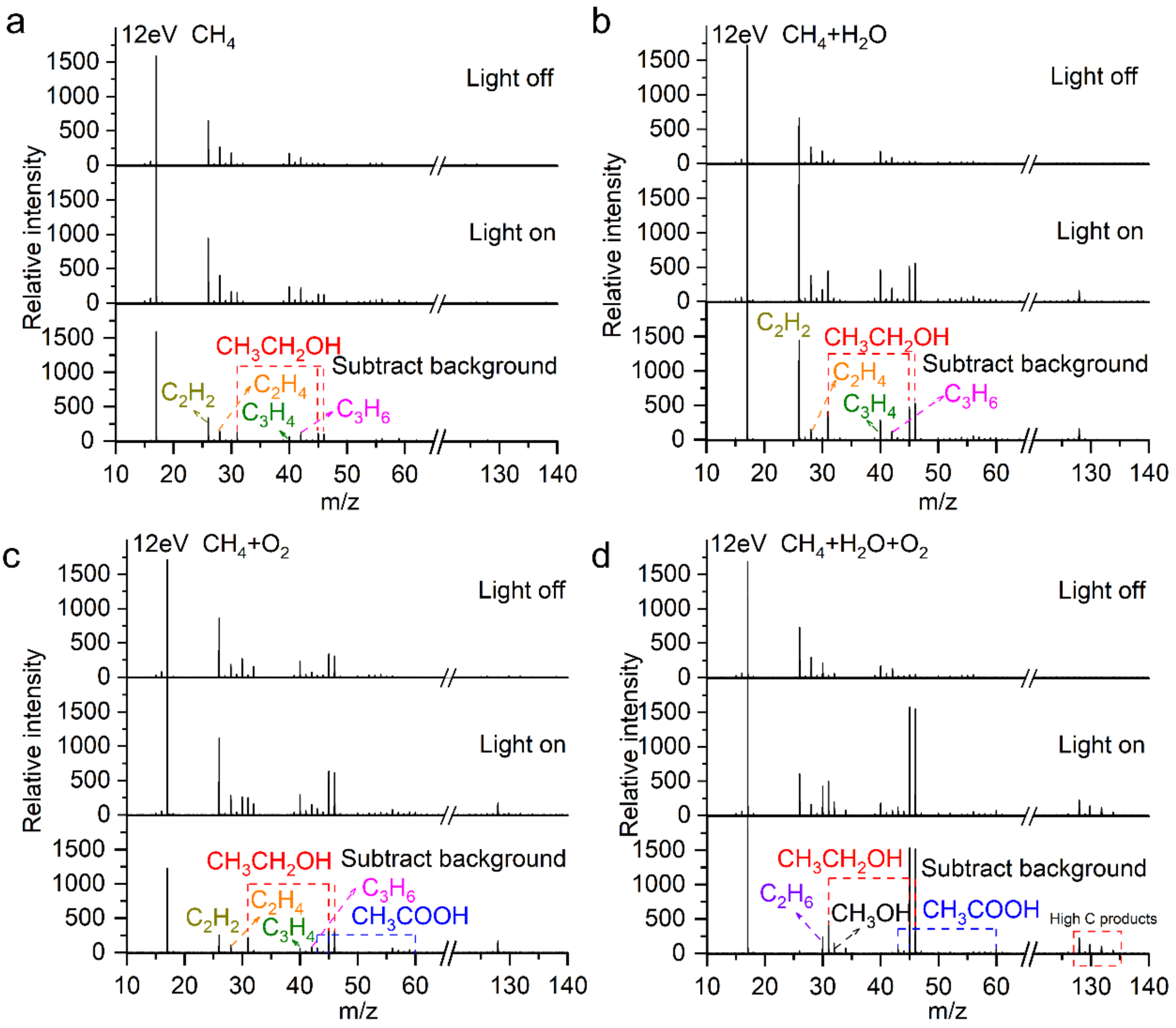

Fig. S12 SR-PIMS spectra of CTF-1 with a photon energy of 12 eV in (a) CH_4, (b) CH_4+H_2O, (c) CH_4+O_2, and (d) CH_4+H_2O+O_2.

Supplementary Note to Fig. S12.

In-situ synchrotron radiation photoionization mass spectrometry (SR-PIMS), which allows for the detection of various gas-phase intermediates and products with high sensitivity, was used to analyse the products formed from photocatalytic methane oxidation by CTF-1. The photon energy was firstly fixed at 12 eV. In pure CH_4 atmosphere, C_2H_2, C_2H_4, C_3H_4 and C_3H_6 are observed at m/z ratios of 26, 28, 40, and 42, respectively (Fig. S12a). In the meantime, a trace amount of C_2H_5OH is detected at m/z ratios of 31, 45 and 46, likely due to a trace amount of O_2 residue in the purged reactor or adsorbed on the surface of CTF-1. When additional water vapour is introduced into CH_4, similar products are detected to those observed in pure CH_4 (Fig. S12b). However, the intensities of the MS signals are stronger, suggesting that H_2O is a promoter during the photocatalytic partial oxidation of methane. When CH_4 and O_2 are introduced simultaneously, a low concentration of CH_3COOH is observed at m/z ratios of 43, 45 and 60, apart from the products detected in the CH_4 or (CH_4+H_2O) environment (Fig. S12c). This is in agreement with our assumption that O_2 promotes the formation of products that cannot be detected using FID-GC. With the co-existence of CH_4, H_2O and O_2, the intensities of the peaks related to C_2H_5OH are significantly enhanced, compared to those in CH_4, CH_4+H_2O or CH_4+O_2 (Fig. S12d).

This indicates that both O_2 and H_2O are indispensable for the partial oxidation of methane to ethanol and is highly consistent with the performance analysis in Fig 1. A new signal with a m/z ratio of 30 appears, which originates from the production of C_2H_6. This is also consistent with the hypothesis of C_2H_6 as the major intermediate of this photocatalytic process. CH_3COOH is obtained, similar to that in CH_4+O_2. A small amount of CH_3OH is discovered at m/z of 32. C_2H_2, C_2H_4, C_3H_4 and C_3H_6, which are present in the products of the previous conditions, are not detected. It is also noted that serval signals at m/z ratios of 128, 130, 132 and 134, could originate from long-chain products (Table S2) that cannot be detected using GC-FID only.

Table S3 Possible long-chain products generated during photocatalytic methane conversion by CTF-1.

Entry	m/z			
	128	130	132	134
1	$C_5H_4O_4$	$C_5H_6O_4$	$C_4H_4O_5$	$C_4H_6O_5$
2	$C_6H_8O_3$	$C_6H_{10}O_3$	$C_5H_8O_4$	$C_5H_{10}O_4$
3	$C_7H_{12}O_2$	$C_7H_{14}O_2$	$C_6H_{12}O_3$	$C_6H_{14}O_3$
4	$C_8H_{16}O$	$C_8H_{18}O$	$C_7H_{16}O_2$	$C_9H_{10}O$
5		$C_{10}H_{10}$	$C_{10}H_{12}$	$C_{10}H_{14}$

It can be noted that the long-chain products could be C_5-C_9 oxygenates or C_{10} hydrocarbons.

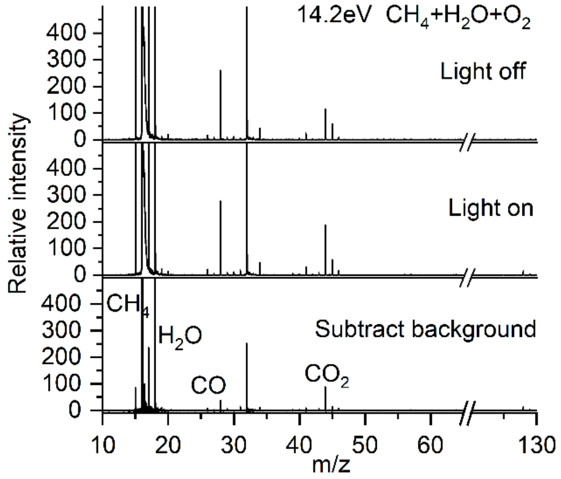

Fig. S13 SR-PIMS spectra of CTF-1 with a photon energy of 14.2 eV in CH_4+H_2O+O_2.

Supplementary Note to Fig. S13.

The photon energy was fixed at 14.2 eV to detect products with a higher ionisation energy. The signal at m/z of 44 originates from the overoxidation product CO_2. A trace amount of CO is also detected at m/z of 28 in the spectrum with subtracted backgournd.

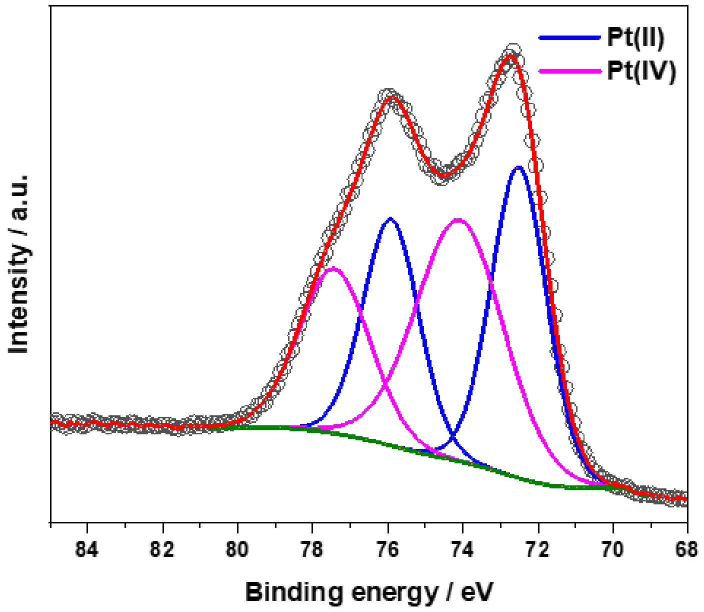

Fig. S14. Pt 4*f* XPS spectrum of 3 wt.% PtO_x/CTF-1.

Supplementary Note to Fig. S14.

Two peaks located at *ca.* 73 eV and 76.5 eV represent the Pt $4f_{7/2}$ and Pt $4f_{5/2}$, respectively.[9] According to the peak integration, Pt species are Pt (II) and Pt (IV) states. The Pt (II) to Pt (IV) ratio is nearly 1:1.

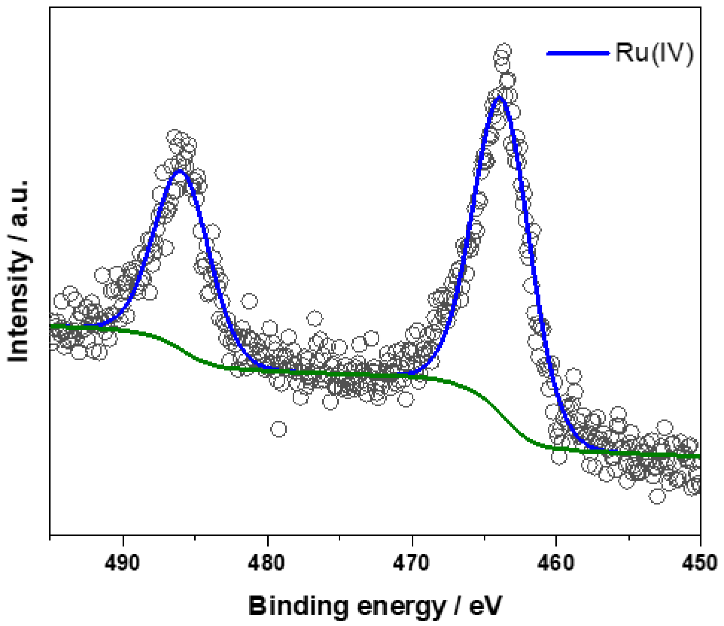

Fig. S15. Ru 3*p* XPS spectrum of 3 wt.% RuO_x/CTF-1.

Supplementary Note to Fig. S15.

Two peaks located at *ca.* 464 eV and 486 eV represent the Ru $3p_{3/2}$ and Ru $3p_{1/2}$, respectively.[10] The observed Ru species are Ru^{4+} (RuO_2) according to the peak fitting.

Table S4 Performance comparison. Methane-to-ethanol conversion over new photocatalyst CTF-1, and all g-C_3N_4 based catalysts synthesised in the current study and relevant recently-published literatures, inlcuding reaction condutions and key criteria of methane conversion, ethanol yield, ethanol selectivity, stability, apparent quantum efficiency, as well as reaction mechanism.

Catalyst	Reaction (including light source or light intensity)	CH_4 conversion /%	EtOH yield / µmol h^{-1} (or µmol h^{-1} g^{-1})	EtOH Selectivity / %	Stability /h	Mechanism	AQY / %
PtO_x-CTF-1 (This work)	Flow, $CH_4+O_2+H_2O\rightarrow C_2H_5OH$, 365 nm LED, 100 mW cm^{-2}, 1 g catalyst	2.3	167.6 (167.6)	79.6	12	Methane to ethane then to ethanol	9.4
CTF-1 (This work)	Flow, $CH_4+O_2+H_2O\rightarrow C_2H_5OH$, 365 nm LED, 100 mW cm^{-2}, 1 g catalyst	1.7	122.4 (122.4)	78.6	50	Methane to ethane then to ethanol	6.9
g-C_3N_4 (This work)	Flow, $CH_4+O_2+H_2O\rightarrow C_2H_5OH$, 365 nm LED, 100 mW cm^{-2}, 1 g catalyst	0.6	29.2 (29.2)	46.1	/	Methane to ethane then to ethanol	1.7
Cu/g-C_3N_4[11]	Batch, $CH_4+H_2O\rightarrow C_2H_5OH$, 500 W Xe lamp, 20 mg catalyst	/	0.42 (21)	53.8	24	Methane to methanol then to ethanol	/
CeO_2[12]	Batch, $CH_4+H_2O\rightarrow C_2H_5OH$,300 W Xe lamp, AM1.5, 100 mW cm^{-2}, 2 mg catalyst	0.02	0.023 (11.5)	91.5	/	Oxygen defects dominated activity	0.3
P-g-C_3N_4[13]	Batch, $CH_4+O_2+H_2O\rightarrow C_2H_5OH$, 300 W Xe lamp, 5 mg catalyst	0.06	0.255 (51)	62.7	4	Methane to methanol then to ethanol	/
Vac_N-g-C_3N_4[14]	Batch, $CH_4+O_2+H_2O\rightarrow C_2H_5OH$, 300 W Xe lamp,	0.16	5.6 (280)	85.1	/	Methane to methanol then to ethanol	/

	100 mW cm^{-2}, 20 mg catalyst						
Fe (III) encapsulated with g-C_3N_4[15]	Batch, CH_4+H_2O→ C_2H_5OH, 300 W Xe lamp, 420 nm filter, 7.5 mg catalyst	0.08	1.29 (171)	86.4	6	Methane to methanol then to ethanol	/

Supplementary Note to Table S4:

The direct transformation of methane into oxygenates with high selectivity under mild conditions, one of the holy grails in catalysis, is challenging because catalysts capable of activating the strong primary C-H bonds in methane can typically easily convert the initial products (such as ethanol) into thermodynamically more stable CO or CO_2. This conversion-selectivity trade-off means that higher methane conversions achieved by using stronger oxidation conditions result in lower selectivities for the desired product. This is illustrated by the g-C_3N_4 based photocatalysts that have achieved notable ethanol production rates in terms of "µmol per gram per hour" during the early stages of methane conversion, but with high H_2O_2 oxidant levels found to cause indiscriminate overoxidation of intermediates to low-value products e.g. CO_2[14]. Our photocatalyst design with separate sites for methane activation and oxidant generation, and proceeding via a distinct mechanistic pathway that involves ethane as intermediate, improves the conversion-selectivity trade-off that can be achieved.

While catalysis research typically focuses on activity and selectivity, productivity is important when aiming to develop industrial processes and requires a careful evaluation of catalysts' stability[16,17]. Characterizing the intrinsic activity of photocatalysts is particularly challenging because photocatalytic conversion can only occur where the photocatalyst is able to absorb photons of an appropriate wavelength. Directly comparing photocatalysts on the basis of absolute product formation rates determined in different studies using different reactors can therefore be as unreliable as comparisons on the basis of measured product formation rates normalized by catalyst weight or comparisons on the basis of measured apparent quantum yields (AQYs): these performance metrics are all influenced by factors such as the reactor type (batch or flow), reaction pressure and

temperature, flow rates, reactants' ratio, light intensity, catalyst loading, fraction of catalyst irradiated by light, etc. And although AQY is widely recognised as a particularly robust criterion to determine the performance of a photocatalyst[18,19], AQY values can be affected by catalyst loading (if the number of catalyst sites accessible to photons isn't evaluated), scattering of light in the reactor and the wavelength range of the irradiating light (https://www.perfectlight.com.cn/solution/mobile-phase/30.html).

The table lists known methane-to-ethanol photocatalysts and their ethanol formation rates in the widely used[18,21-23] unit of μmol h^{-1} and also in μmol $h^{-1}g^{-1}$ (the latter, despite commonly used in thermal catalysis, less reliable for evaluating photocatalysis as explained above and the documented[20]). Where available, we also list AQY values. We emphasize again, however, that reliable performance comparisons are difficult because factors that can significantly impact all these performance metrics include the catalyst loading, reactor size and configuration, temperature, pressure, irradiation source and area, etc, which should be clearly stated and ideally standardized as far as possible to enable meaningful cross-comparisons between different studies and photocatalysts and thereby more efficient and informed development.

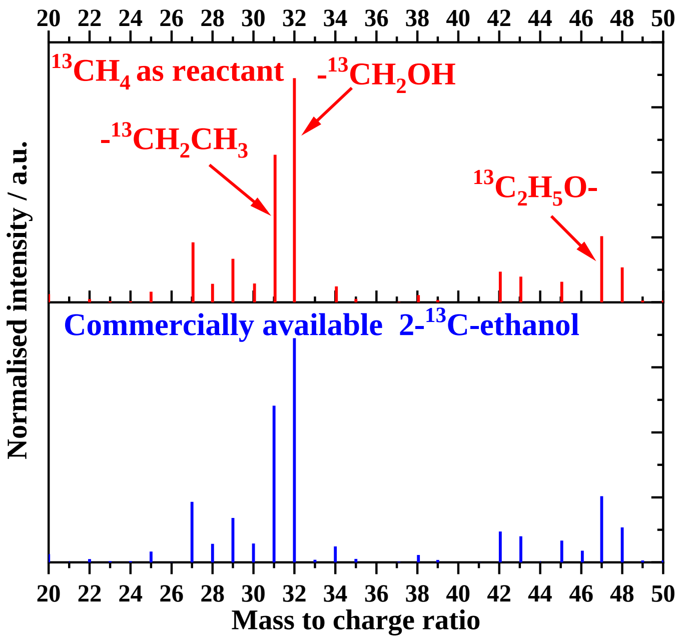

Fig. S16. Mass spectra of the major product ethanol.

Supplementary Note to Fig. S16.

Ethanol produced when using $^{13}CH_4$ (> 99.5 atom % Sigma-Aldrich) + $^{16}O_2$ + $H_2{}^{16}O$ (top panel) in this work and the commercially available 2-^{13}C-ethanol from Sigma-Aldrich (99 atom % ^{13}C) (bottom panel) nearly shows the same peaks, indicating that both carbon atoms in ethanol are generated from methane.

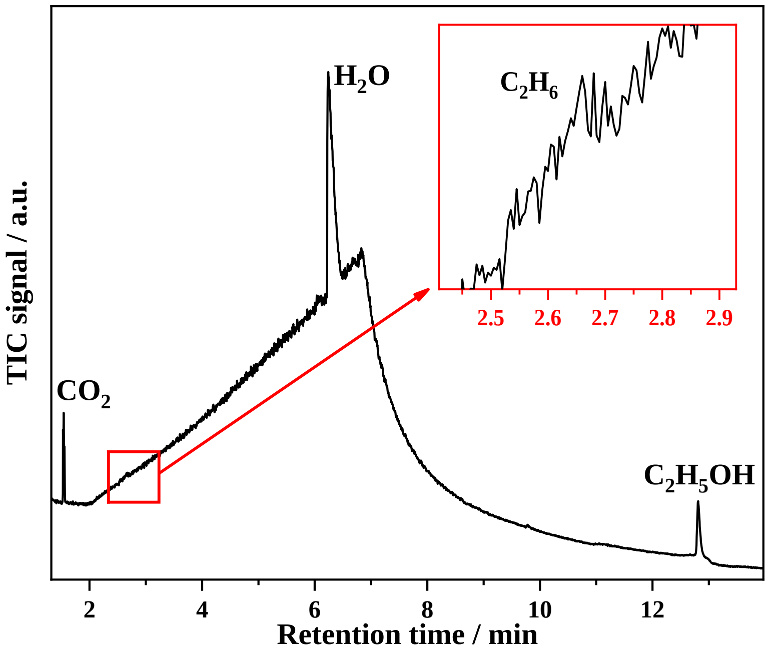

Fig. S17. Total Ion Chromatogram spectrum of all detectable reactants and products.

Supplementary Note to Fig. S17.

The total ion chromatogram (TIC) spectrum presents four evident peaks. The first peak located at *ca.* 1.7 min is assigned to CO_2. After that, although the baseline begins to rise, there is a clear signal at *ca.* 6.5 min assignable to water. The last peak at *ca.* 12.9 min is assigned to the product ethanol, confirmed by the associated mass spectrum. A small peak at *ca.* 2.65 min as shown in the insert is identified as ethane by the mass spectrum (Fig. S19), which is a likely intermediate during the reaction thought the signal is very weak. No signals of other reactants (*e.g.* O_2, N_2 and CH_4) are detected as those were purged out before the detector operation, because the concentrations of these feedstocks are very high and can result in damage to the ion source.

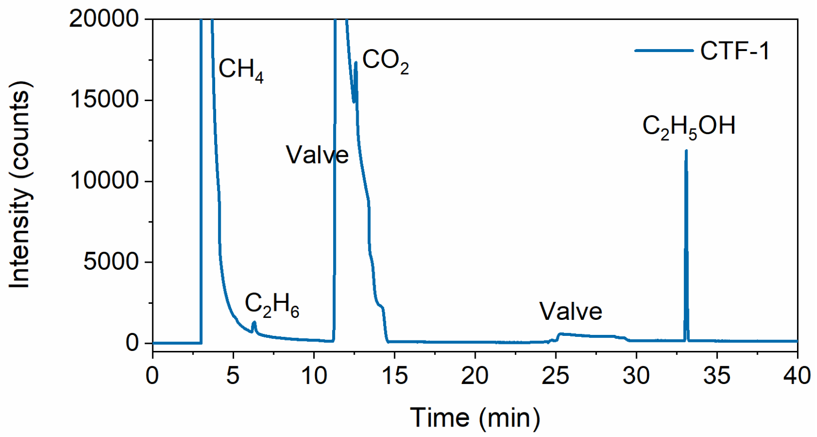

Fig. S18 GC-FID spectrum of the products formed in photocatalytic partial oxidation of methane by CTF-1.

Supplementary Note to Fig. S18.

The products obtained from photocatalytic methane oxidation by CTF-1 were evaluated using GC-FID. The main product C_2H_5OH displays the strongest peak with a retention time of 33.1 min. The intermediate C_2H_6 and overoxidation product CO_2 are observed at 6.3 and 12.6 min, respectively. The two large peaks at 11.3 and 25.2 min originates from the valve switch. The yield of the products is quantified by the integrated area of the corresponding peaks. The production rates of C_2H_5OH, C_2H_6, and CO_2 are calculated to be 121.8, 2.6 and 34.3 μmol/h, respectively.

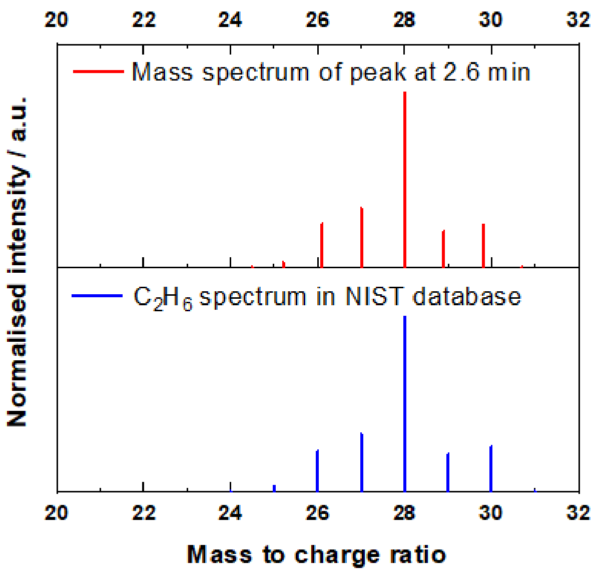

Fig. S19. Mass spectra of peak at 2.65 min shown in Fig. S13 (top panel) and the standard mass spectrum (electron ionisation) of ethane from NIST database (NIST MS number 23) (bottom panel).

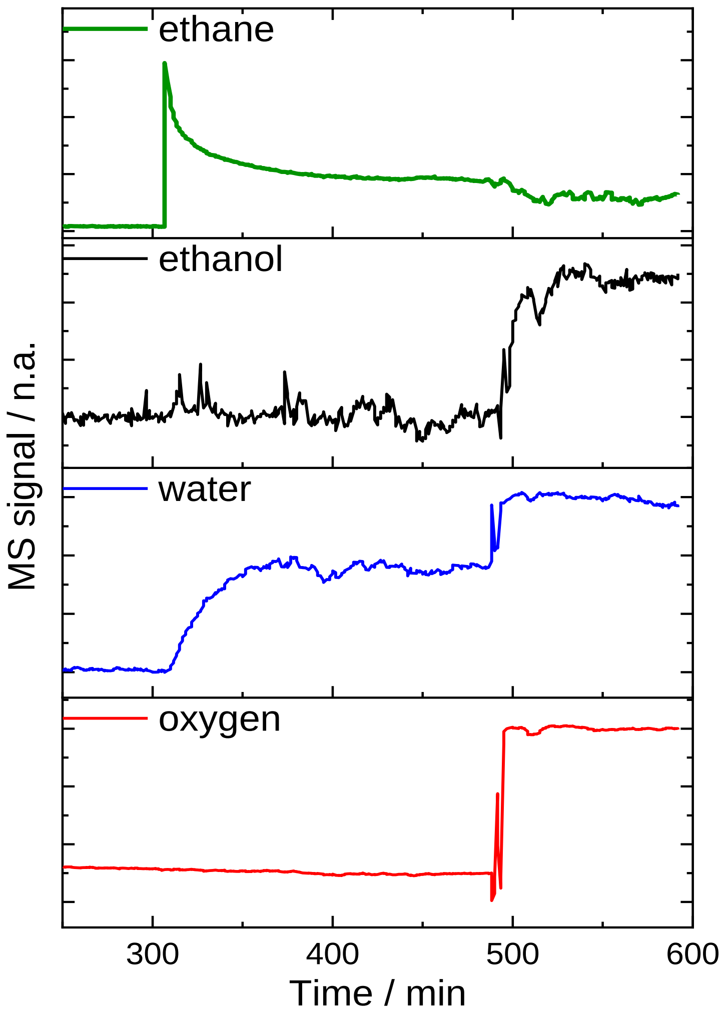

Fig. S20. Mass spectral responses of reactants and products. Ethane (green line, *m/z* = 30), ethanol (black line, *m/z* = 46), water (blue line, *m/z* = 18), oxygen (red line, *m/z* = 32) of the outlet gas during the selective photocatalytic oxidation of methane. Reaction conditions: gas flow rate 40 mL min^{-1}, 365 nm LED irradiation.

Supplementary Note to Fig. S20.

During the reaction, the CTF-1 catalyst was purged with methane under LED light irradiation. As indicated by the green line in the top panel, there was no ethane generation without water or oxygen during the first 310 min. When water was added at 310 min (blue line), ethane was firstly generated and then decreased for 10 minutes, which may be attributed to the surface poisoning by the adsorbed protons in the absence of O_2 (CH_4 + H_2O_{diss}(surf) → H_2O(g) + CH_3(surf) + H(surf), Δ_H CTF = +84 kJ/mol). When oxygen gas was added to the system after 490 minutes (red line) to regenerate the photocatalyst surface by capturing the adsorbed protons, ethanol was generated simultaneously (black line).

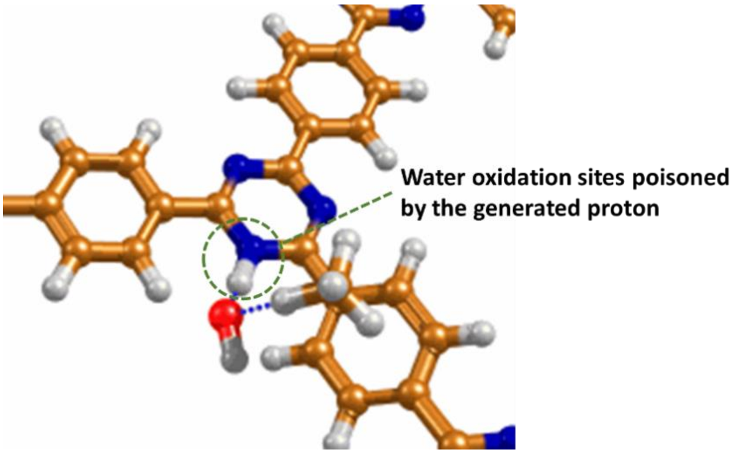

Fig. S21. Formation of methyl radical in the presence of water over CTF-1. The blue, brown, red and gray denote N, C, O and H, respectively.

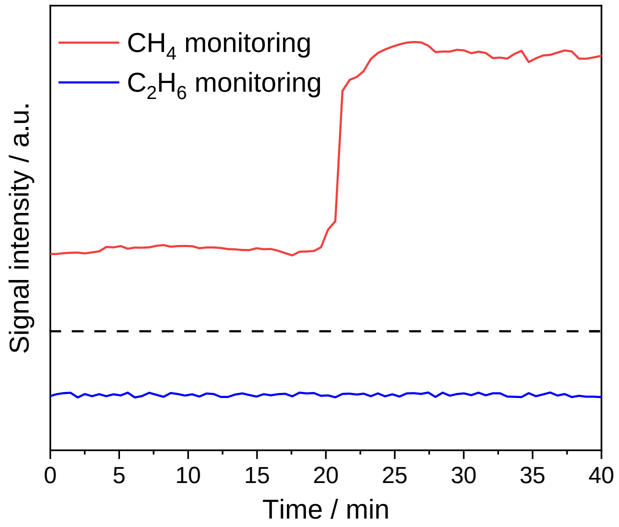

Fig. S22. Mass spectra of CH_4 and C_2H_6 under the dark condition without a catalyst.

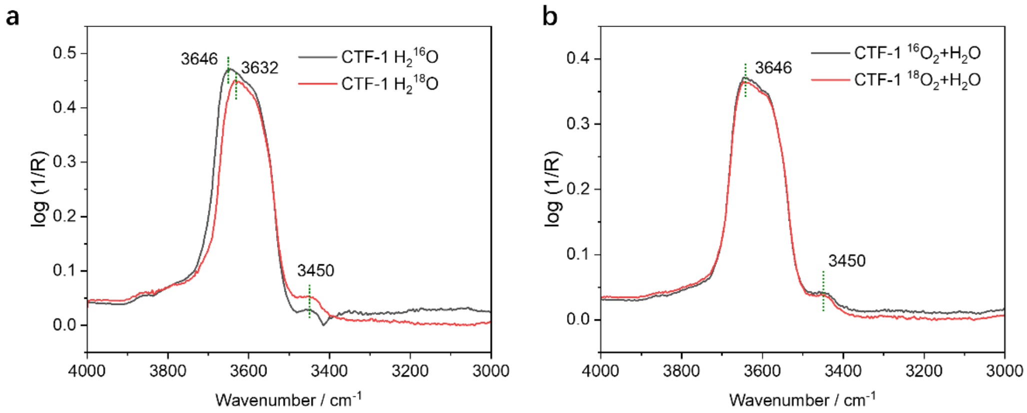

Fig. S23 DRIFTS spectra of (a) H_2O and $H_2{}^{18}O$ adsorptions on CTF-1 (P_{H_2O} = 3 kPa), and (b) O_2+H_2O and $^{18}O_2$+H_2O co-adsorptions on CTF-1 (total pressure: 1 atm; P_{H_2O} = 3 kPa) at RT.

Supplementray Note to Fig. S23.

The isotopic O_2 and H_2O adsorption measurements by DRIFTS were carried out to monitor the exchange. Isotopic $H_2{}^{18}O$ adsorption spectra of CTF-1 are shown in Fig. S23a. The band at 3646 cm^{-1} is ascribed to OH vibration in H_2O and a shift of the band to 3632 cm^{-1} is observed when $H_2{}^{18}O$ is used. The shoulder band at 3450 cm^{-1} originates from the N-H structure in CTF-1, no shift of this band is detected as only O is isotopically labelled. Then, the adsorption of H_2O in the presence of O_2 gas was carried out. No visible difference in the band position of the DRIFTS spectra (Fig. S23b) is observed when O_2 is replaced by $^{18}O_2$. This indicates that there is no detectable exchange between O_2 and H_2O in the reactant.

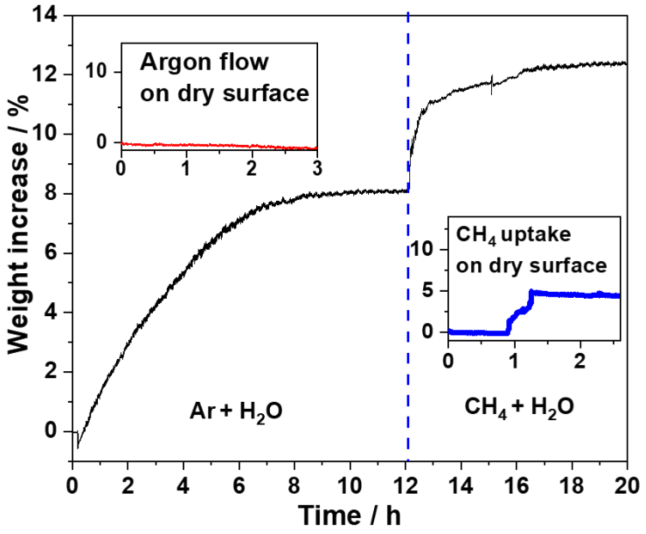

Fig. S24. Argon, methane and water adsorption on CTF-1 surface under ambient conditions (25 ºC, 1 bar).

Supplementary Note to Fig. S24.

There is no weight increase indicating no argon adsorption on CTF-1 under such conditions (the left insert). Thereafter, water molecules were carried by argon to the reactor to investigate the water adsorption ability of CTF-1 (black line 0-12 h). After that argon was replaced by methane to study the methane adsorption on humidified CTF-1 surface (black line 12-20 h). Compared to the methane adsorption on dry CTF-1 surface (blue line in the right insert), water does not have an obvious influence on methane adsorption as under both cases, weight increase by methane adsorption is very similar, about 5%.

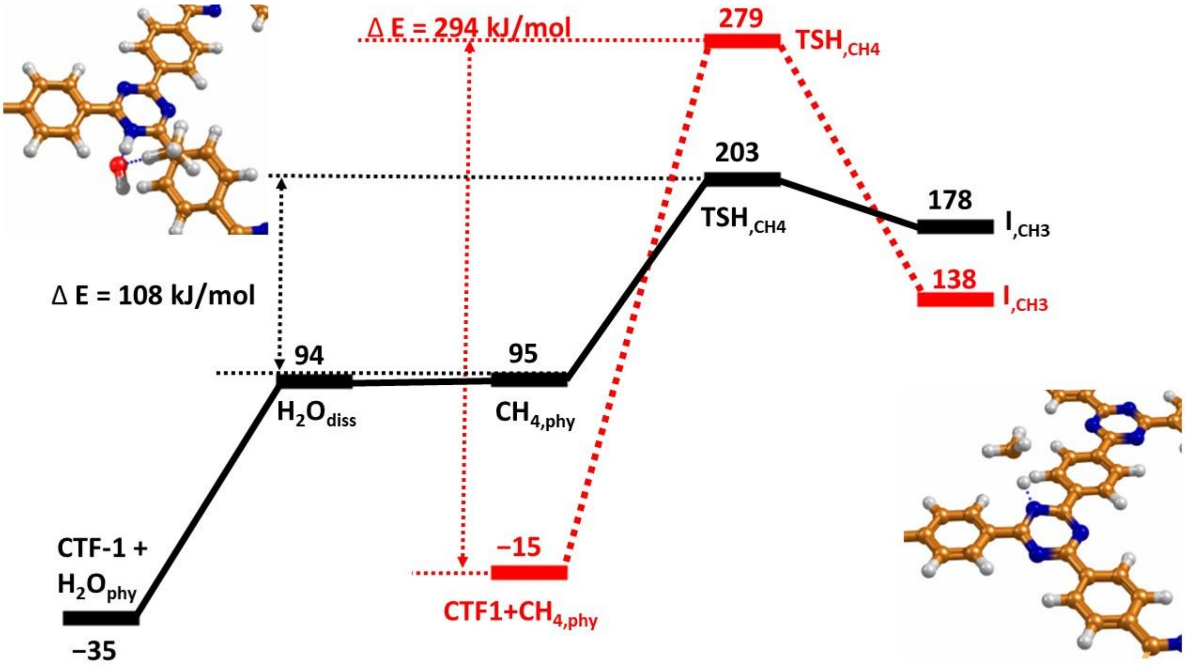

Fig. S25. Methane activation barriers for methane conversion by the CTF-1 polymer with and without water dissociation.

Supplementary Note to Fig. S25.

Energies are given in kJ/mol (C, N, H, and O atoms are displayed in gold, blue, white and red, respectively). Without the dissociation of water on the catalyst surface, the activation energy of the methane molecule is ca. 294 kJ/mol on CTF-1. While after water dissociation on the surface of CTF-1, the barrier for the methane activation is only about 108 kJ/mol, indicating the key role of dissociated water on the surface of CTF-1 to accelerate the reaction. The blue, brown, red and gray denote N, C, O and H, respectively.

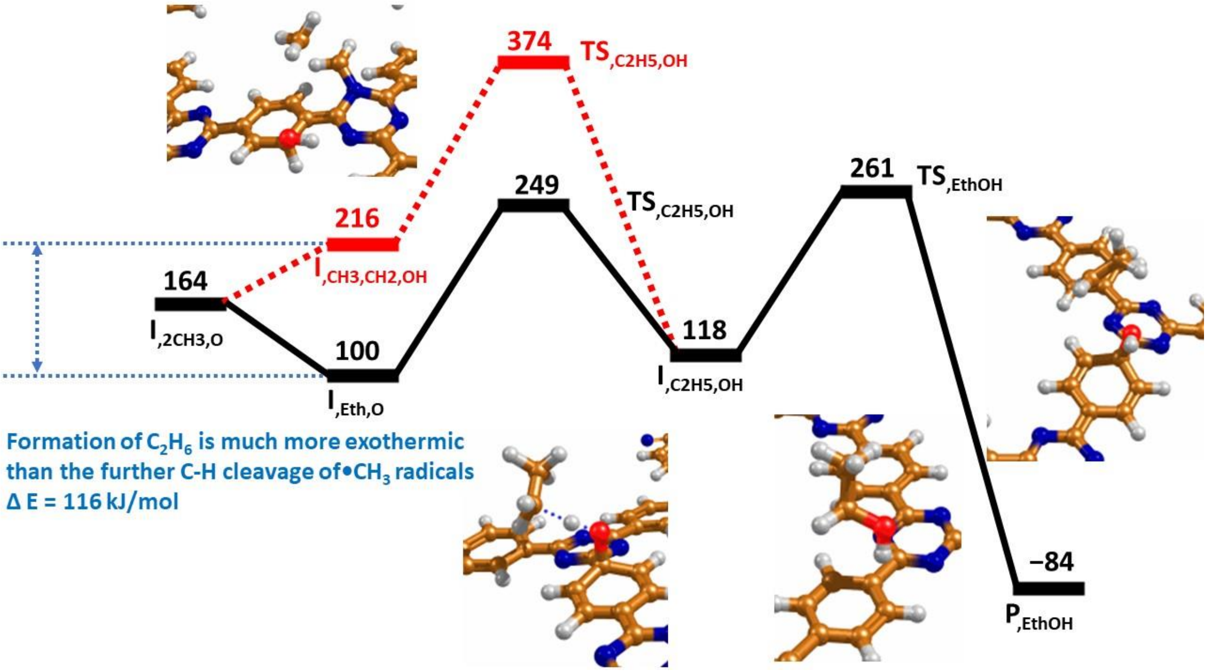

Fig. S26 Reaction pathways and energy profile for the formation of ethanol from two surface-bound methyl radicals over CTF-1. Reaction energies are given in kJ/mol^{-1} (C, N, H, and O atoms are displayed in gold, blue, white and red, respectively).

Supplementary Note to Fig. S26.

The formation of ethane is 116 kJ/mol more exothermic than the further C-H cleavage of methyl radicals to form C_2H_5 radicals. Therefore, two methyl radicals prefer to recombine first to form an ethane molecule.

There are two transient steps with similar energy barriers competing to be the rate-limiting step for the formation of ethanol. The ethane activation step (**TS,$_{C2H5,OH}$**) and the ethanol formation step (**TS,$_{EthOH}$**) show barriers of 149 and 143 kJ/mol respectively, both being considerably higher than the 108 kJ/mol associated with the methane activation (see Fig S25). Thus, the partial oxidation of the intermediate, ethane, is more favourable under O_2 atmosphere The blue, brown, red and gray denote N, C, O and H, respectively.

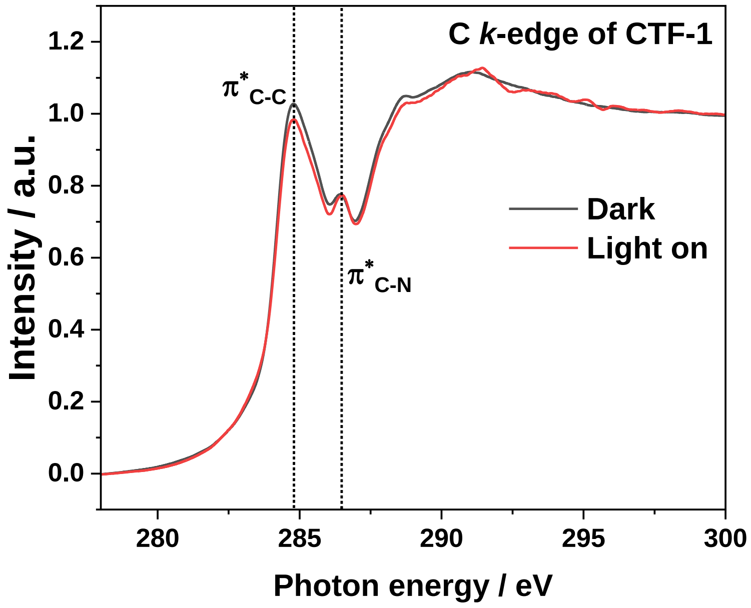

Fig. S27. C *k*-edge NEXAFS spectra of CTF-1 in the presence or absence of LED light (320 nm) irradiation, respectively.

Supplementary Note to Fig. S27.

The NEXAFS represents the excitation of electrons from a core level to partially filled or empty states (the conduction band). As shown in Fig. 3B, the bands at 397.1 eV are due to the π transition of N 1*s* →N 2*p* in the triazine unit. The broad band at 403-405 eV was associated with the σ transition of the N atoms. When the sample is irradiated by LED light, the increase in the intensity of the N K-edge spectrum indicates that more electrons are able to be accepted by the N 2*p* orbitals, which implies that hot electrons are transferred away, while holes are accumulated on the N sites of the triazine unit in the presence of LED irradiation. As shown in Fig.S27, the bands at 284.8 and 286.5 eV are due to the π transition of C 1*s*→C 2*p* in the benzene rings (labelled as π*C-C) and the triazine motifs (labelled as π*C-N), respectively. An evident intensity of π*C-C decays under LED light irradiation, while the intensity of π*C-N remains unchanged, indicating that the unoccupied states in the C 2*p* orbitals of the benzene units are reduced under LED light irradiation, i.e. the photo-excited electrons are likely transferred to /accumulated at the carbon sites of the benzene units. This reduces the amount of the excited electrons in π*C-C under the synchrotron radiation, leading to a weaker NEXAFS intensity for the case of LED light irradiation. In total, the comparative NEXAFS results clearly confirm that the hot electrons are able to be transferred to the benzene units, whereas photo-holes accumulate in the triazine motifs in the CTF-1 sample.

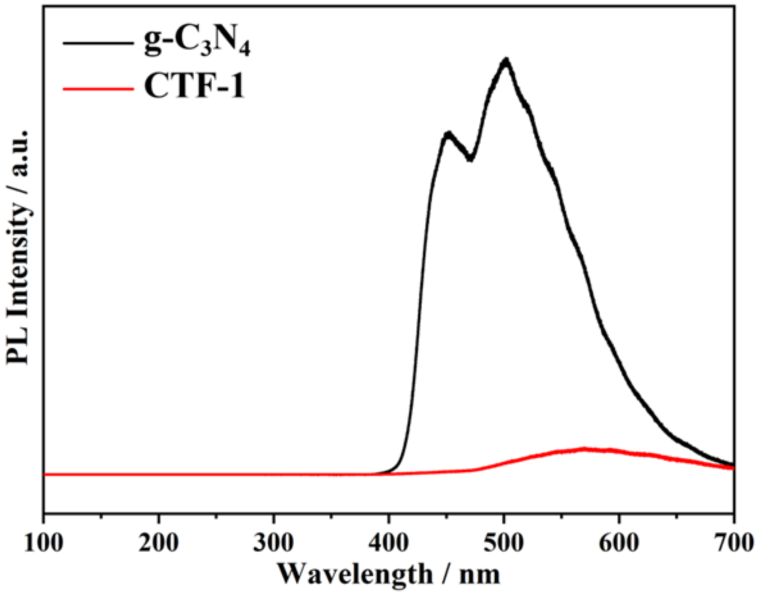

Fig. S28. Photoluminescence (PL) spectra of g-C_3N_4 and CTF-1.

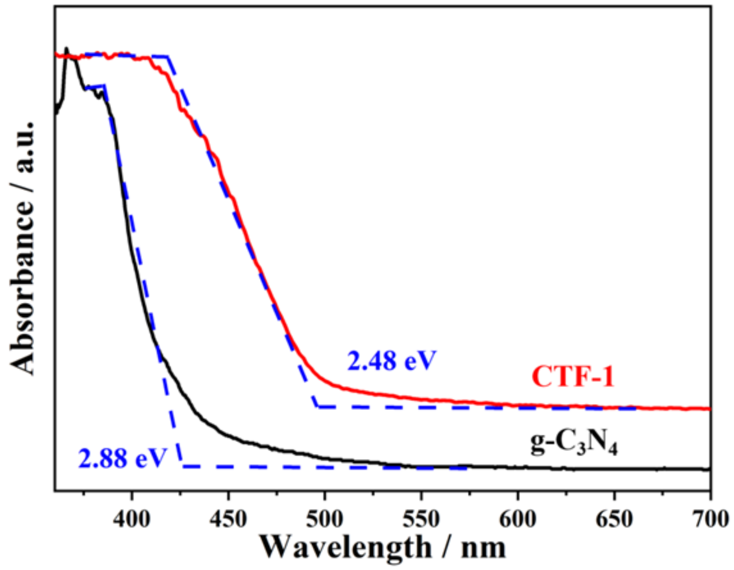

Fig. S29. UV-Vis absorption spectra of g-C_3N_4 and CTF-1.

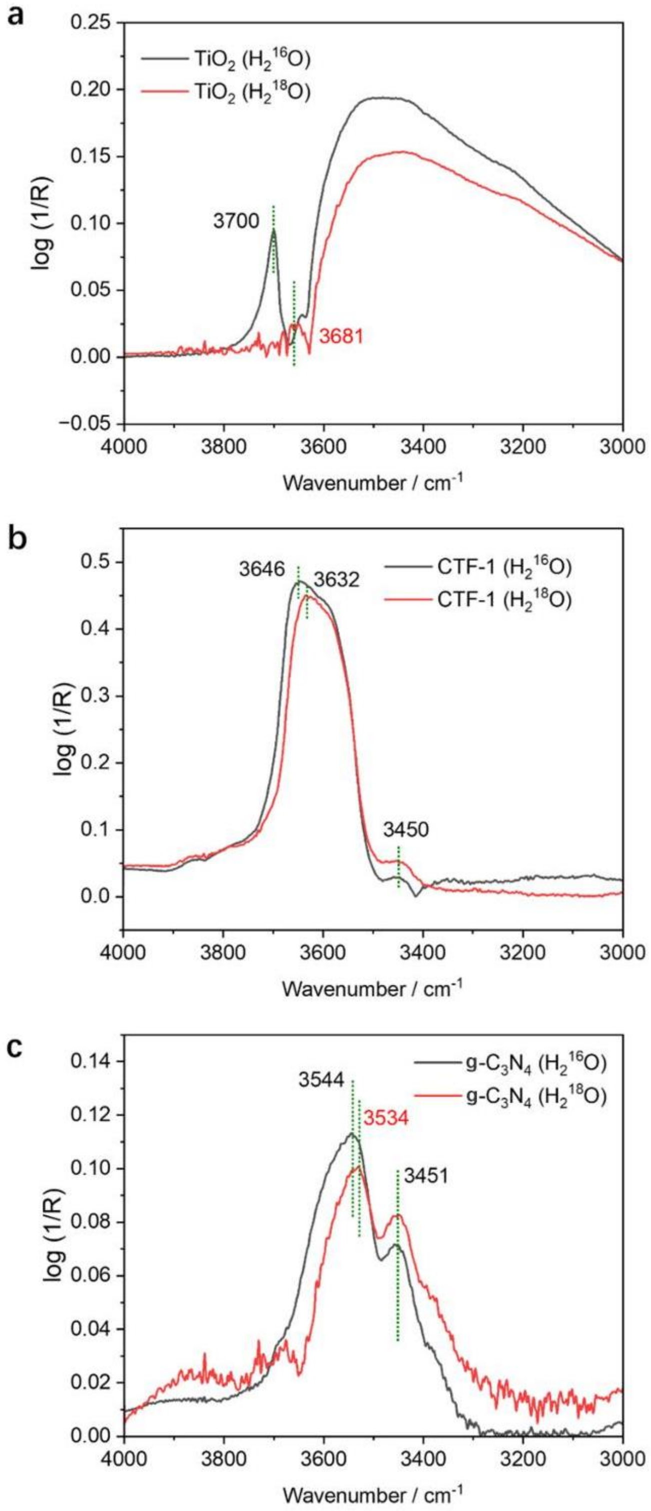

Fig. S30. DRIFTS spectra of H_2O adsorption on (A) TiO_2, (B) CTF-1 and (C) g-C_3N_4 at RT (P_{H2O}=3 kPa). $H_2{}^{16}O$ and $H_2{}^{18}O$ are presented by black and red lines.

Supplementary Note to Fig. S30.

When normal H_2O is replaced by $H_2{}^{18}O$, a band shift was observed in TiO_2, CTF-1 and g-C_3N_4, respectively, from 3700 to 3681 cm^{-1}, 3646 to 3632 cm^{-1}, and 3544 to 3534 cm^{-1}. This indicates that the bands in the range from 3500 to 3700 cm^{-1} are resulted from the adsorbed water. The shoulder band in the spectrum of CTF-1 at 3450 cm^{-1} does not shift, which is ascribed to the N-H vibration mode, as a

similar result (at 3451 cm^{-1}) is also observed on g-C_3N_4. The normal water IR peak on CTF-1 is located at 3646 cm^{-1}, different from that on g-C_3N_4 (3544 cm^{-1}), suggesting that the water adsorption site (or micro-environment) on CTF-1 is different from that on g-C_3N_4. Therefore, the results are consistent with the computational simulation shown in Fig. 3C and Fig. S31.

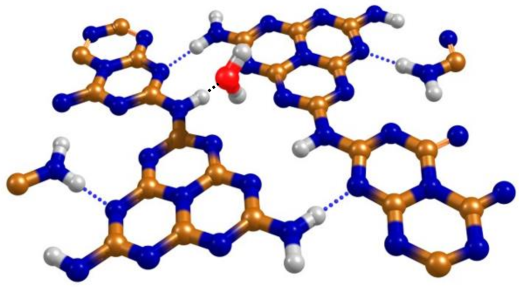

Fig. S31. Calculated water adsorption site on g-C_3N_4. (C, N, H, and O atoms are displayed in gold, blue, white and red, respectively).

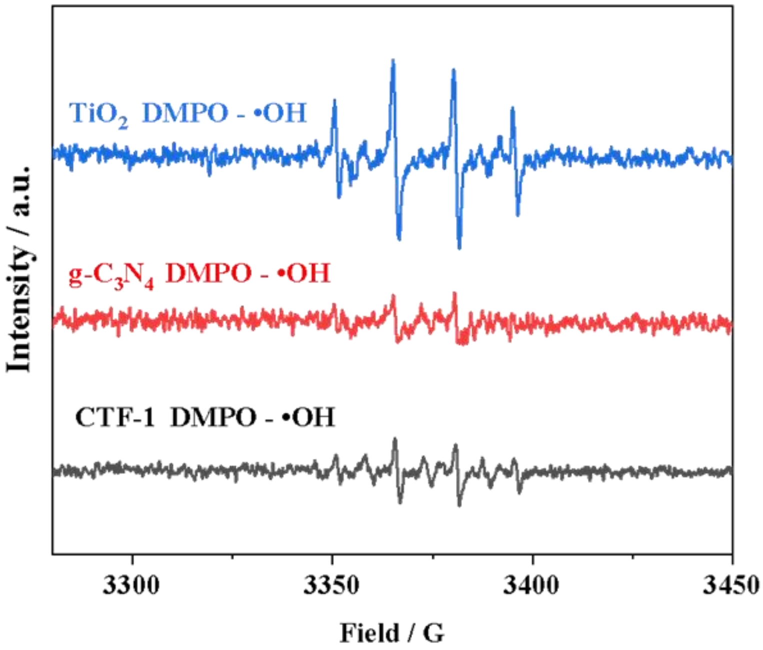

Fig. S32. ***In-situ*** **DMPO-OH˙ spin-trapping EPR spectra over CTF-1, g-C_3N_4 and TiO_2 in water under 90 seconds LED irradiation (λ = 365 nm, 10 W).**

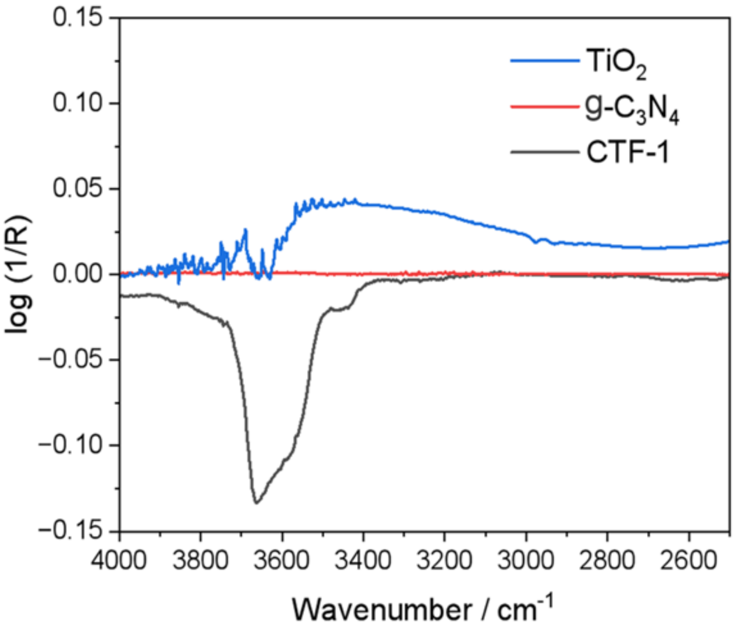

Fig. S33. DRIFTS of H_2O saturated CTF-1, g-C_3N_4 and TiO_2 at RT under light irradiation. The spectra are the subtracted one from that monitored when the light is off. This negative peak observed on CTF-1 indicates efficient desorption of •OH radicals.

Supplementary Note to Fig. S33.

The broad peak observed for the TiO_2 sample shifts from *ca.* 3200 to *ca.* 3400-3500 cm^{-1}, indicating the generation of •OH radicals adsorbed on the surface of TiO_2. In contrast, the CTF-1 sample shows a negative peak at *ca.* 3680 cm^{-1}, suggesting the dissociation of water due to its reaction with photoholes and the detachment of the •OH radicals under light irradiation, which to some extent shed light on the favourable activation of water on CTF-1. On the other hand, g-C_3N_4 does not show any difference in water activation with light irradiation.

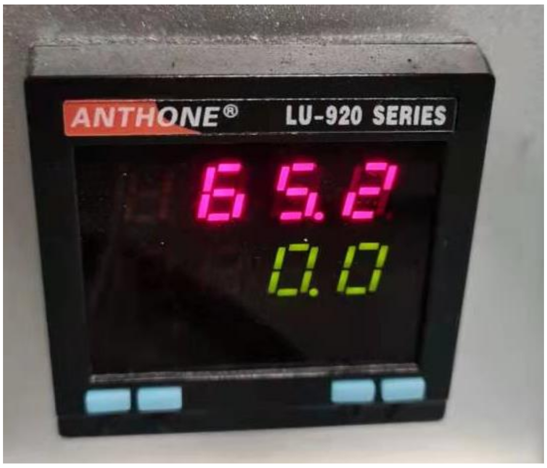

Fig. S34. The monitored temperature of CTF-1 under methane conversion reaction conditions.
Supplementary Note to Fig. S34.
To rule out the possibility that thermal effects caused by light irradiation play a significant role, the surface temperature of the photocatalyst was monitored for a period of 30 min during the photocatalytic methane conversion reaction. The surface temperature of the catalysts vary slightly between 62 ℃ and 67 ℃ under identical experimental conditions.

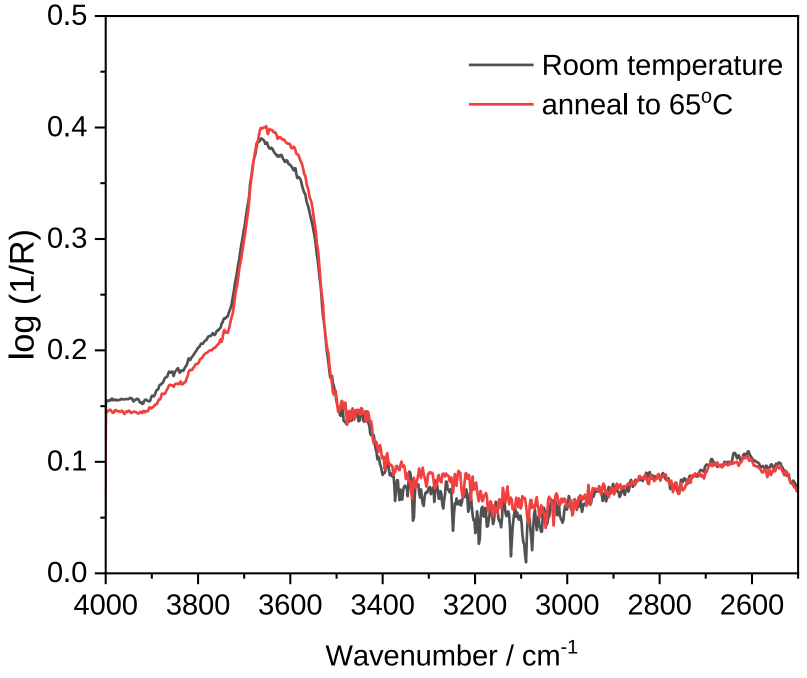

Fig. S35. DRIFTS spectra of CTF-1 in the presence of water vapor at RT and 65 °C under dark conditions.

Supplementary Note to Fig. S35.

The corresponding spectra of CTF-1 in argon and in dark were used as the background. The intensity of the water adsorption peak at 65 °C under dark conditions is similar to that at room temperature, indicating that the negative peak under light irradiation in Fig. S33 is due to water oxidation by photoholes, rather than the catalyst's surface warming by light irradiation.

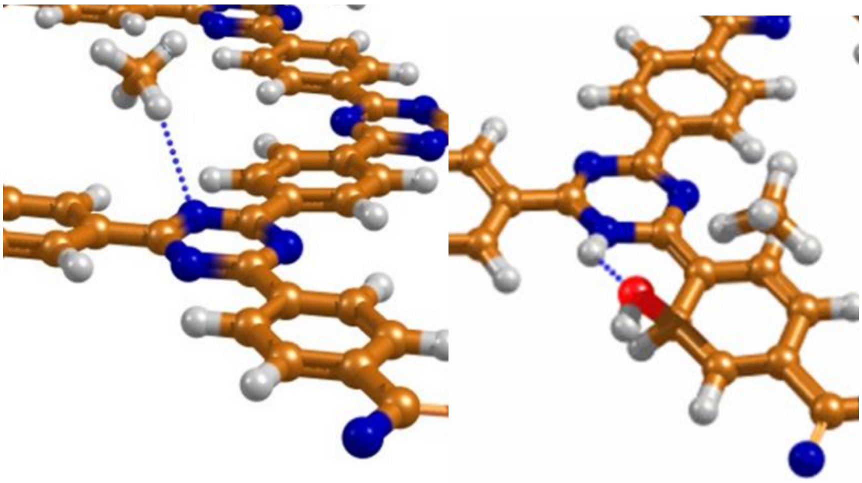

Fig. S36. Calculated methane adsorption sites on CTF-1, (left) physically adsorbed states before water dissociation; (right) physically adsorbed states after water dissociation.

Supplementary Note to Fig. S36.

The corresponding energy for methane sorption without (left) and with the water (right) is 15 and 1 kJ/mol, respectively (C, N, H, and O atoms are displayed in gold, blue, white and red, respectively).

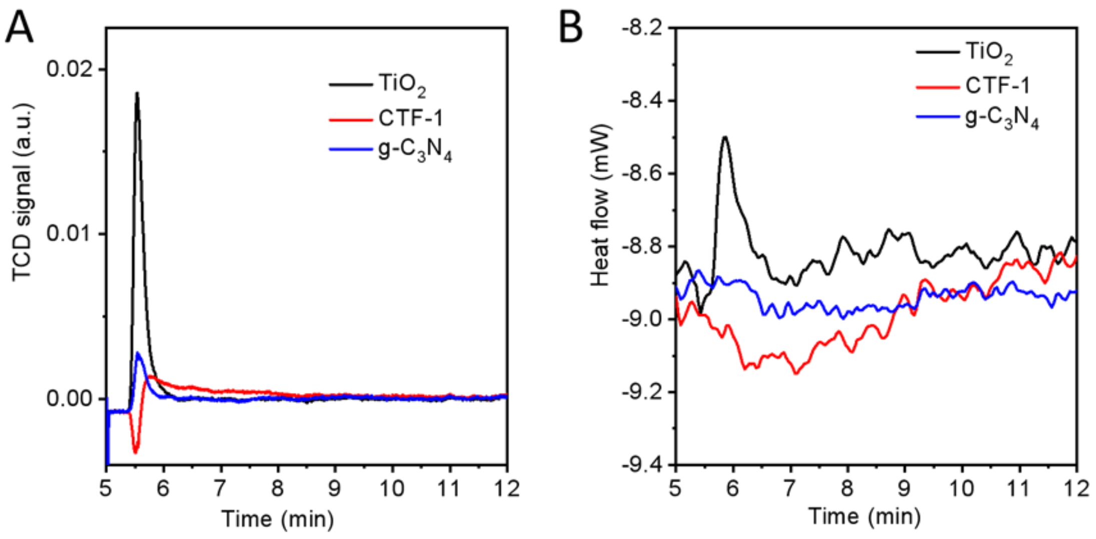

Fig. S37. Isothermal adsorption (A) and calorimetric measurements (B) of O_2 (5% O_2 in He) over 50 mg O_2-saturated CTF-1, g-C_3N_4 and TiO_2 at 173 K.

Supplementary Note to Fig. S37.

Both of CTF-1 and g-C_3N_4 show a smaller amount of O_2 adsorption and smaller energy compared with TiO_2. For CTF-1, negative peaks present on both isothermal desorption and calorimetric measurement at the first few seconds, which is likely owing to the replacement of adsorbed water residual by adsorbed O_2. The sample is degassed thoroughly in helium at high temperature and then decreased to 173 K, thus only adsorbed water can exist in the system, which likely indicates a strong chemisorption of water on CTF-1 surface.

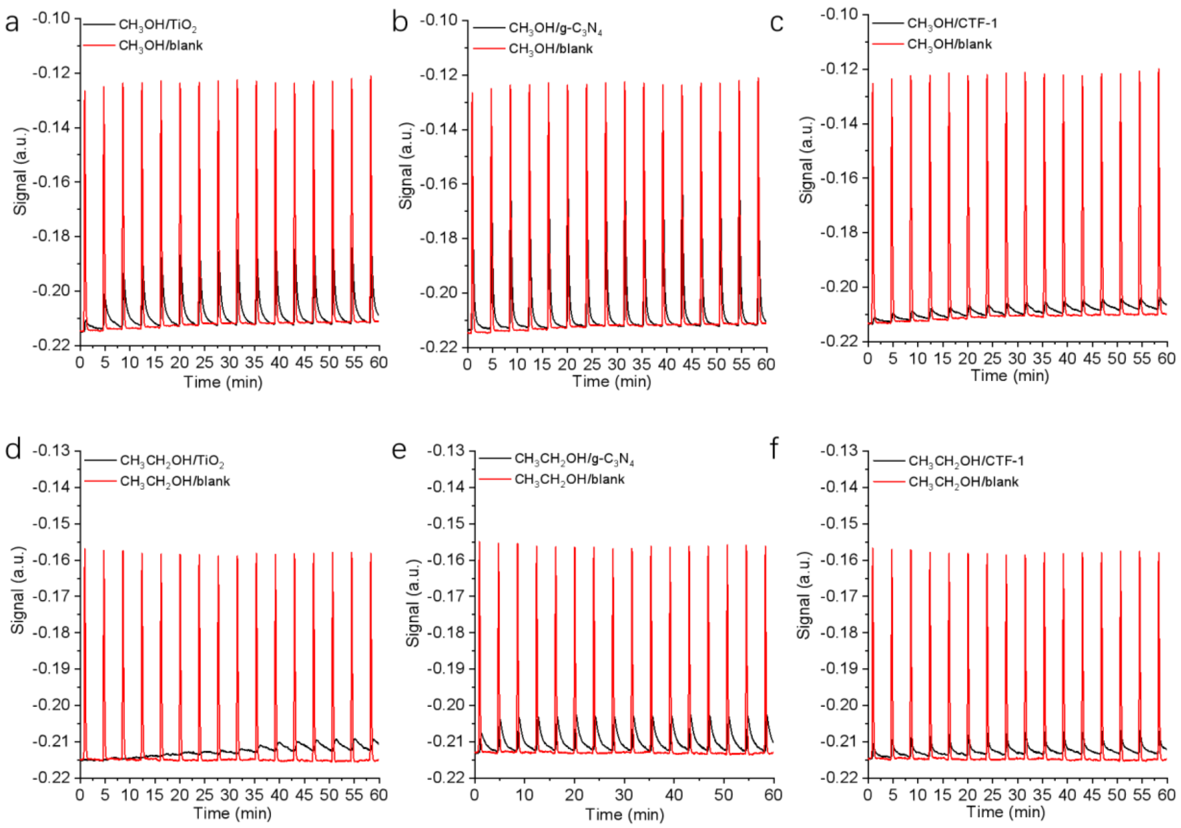

Fig. S38. (a1-a3) TCD signals during CH_3OH pulse adsorption process on various catalysts at 333 K with P(CH_3OH)=27864 Pa. (catalyst amount, TiO_2 0.1g，g-C_3N_4 0.08g, CTF-1 0.2g). (b1-b3) TCD signals during CH_3CH_2OH pulse adsorption process at 333 K with P(CH_3CH_2OH)=13330 Pa. (catalyst amount, TiO_2 0.1 g，g-C_3N_4 0.16 g, CTF-1 0.2 g).

Supplementary Note to Fig. S38.

The difference between the blank curve (red) and the sample curve (black) is attributed to the adsorbed alcohol. The pulse chemisorption is used to measure the irreversible adsorption of methanol and ethanol on three samples.

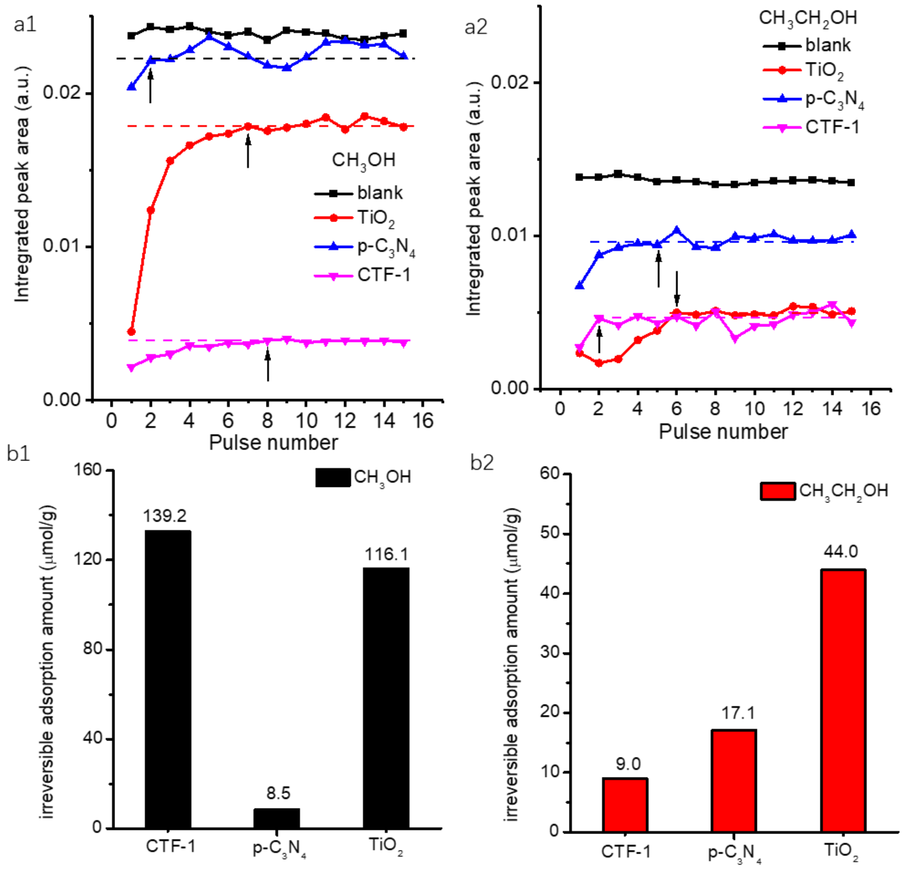

Fig. S39. (a1-a2) The integral peak area as a function of CH_3OH and CH_3CH_2OH pulse adsorption number over various catalysts. (b1-b2) Calculated irreversible adsorption amount (*μ*mol/g) of CH_3OH and CH_3CH_2OH on various catalysts based on the data presented in a1 and a2 where arrows indicate the change from irreversible adsorption to reversible adsorption of the reactants.

Supplementary Note to Fig. S39.

TiO_2 shows a strong adsorption capability for both methanol and ethanol, thus favoring CO_2 production other than alcohols. CTF-1 is able to adsorb methanol at an amount of 139.2 μmol/g, which is much higher than g-C_3N_4 (8.5 μmol/g). The difference in methanol and ethanol adsorption amount on CTF-1 is 130.2. μmol/g, much bigger than that on g-C_3N_4 (8.6 μmol/g). All these indicate a high selectivity to ethanol on CTF-1 can be in part explained by the alcohol adsorption difference.

Table S5. ***In silico* adsorption energies.** The most exothermic adsorption energies calculated for each polymeric photocatalyst. All results are given in kJ/mol.

Adsorbate	ΔE_{ads} of g-C_3N_4	ΔE_{ads} of CTF-1
O	+29.38	+119.49
O_2	−7.91	−28.00
H_2O	−26.83	−35.18
CH_4	−10.48	−15.26
CH_3OH	−30.56	−39.81
C_2H_6	−16.47	−18.12
CH_3CH_2OH	−28.82	−17.99

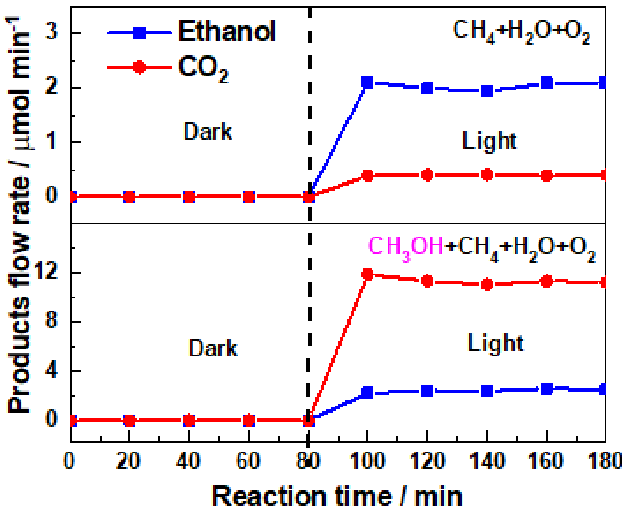

Fig. S40. Online ethanol (blue line) and CO_2 (red line) generation during photocatalytic methane transformation by CTF-1 under identical reaction conditions in the presence (bottom panel) or absence (top panel) of methanol in the feed gas source.

Supplementary Note to Fig. S40.

Reaction conditions: 100 W 365 nm LED irradiation, GHSV= 2000 mL h^{-1}, 16:1 methane (20% methane/Argon) to oxygen (humidified air, 20% oxygen/nitrogen) flow ratio. After introducing methanol into the feed gas, the ethanol generation shows no evident change, but the CO_2 generation rate greatly increases. Thus, methanol is preferentially over-oxidised to CO_2 rather than reacting with methyl radicals to form ethanol on the CTF-1 catalyst, which also indicates that there are different activation sites for ethanol generation and methanol over-oxidation.

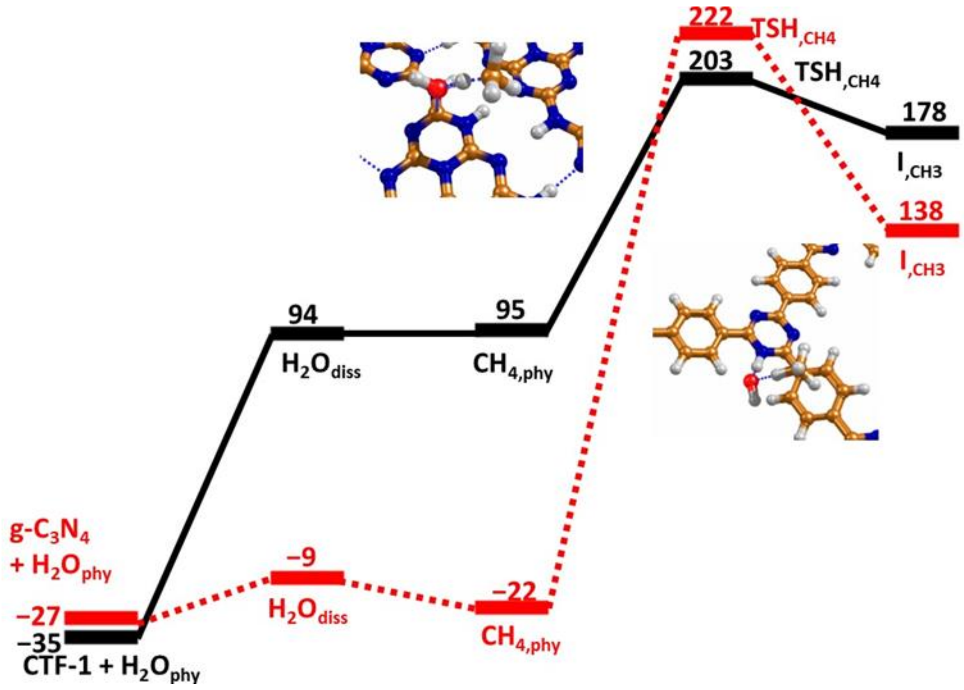

Fig. S41. Reaction energies for methane to methyl radical by dissociated water on CTF-1.

Supplementary Note to Fig. S41.

The modelling result in part indicates that water activation is endothermic on both the surface of g-C_3N_4 and CTF-1. Whilst, OH radical generation is much more endothermic on CTF-1, this drastically decreases the relative barrier of methane activation. Subsequently, methane activation is the rate determine step in g-C_3N_4 with an activation barrier of 244 kJ/mol. Energies are given in kJ/mol (C, N, H, and O atoms are displayed in gold, blue, white and red, respectively).

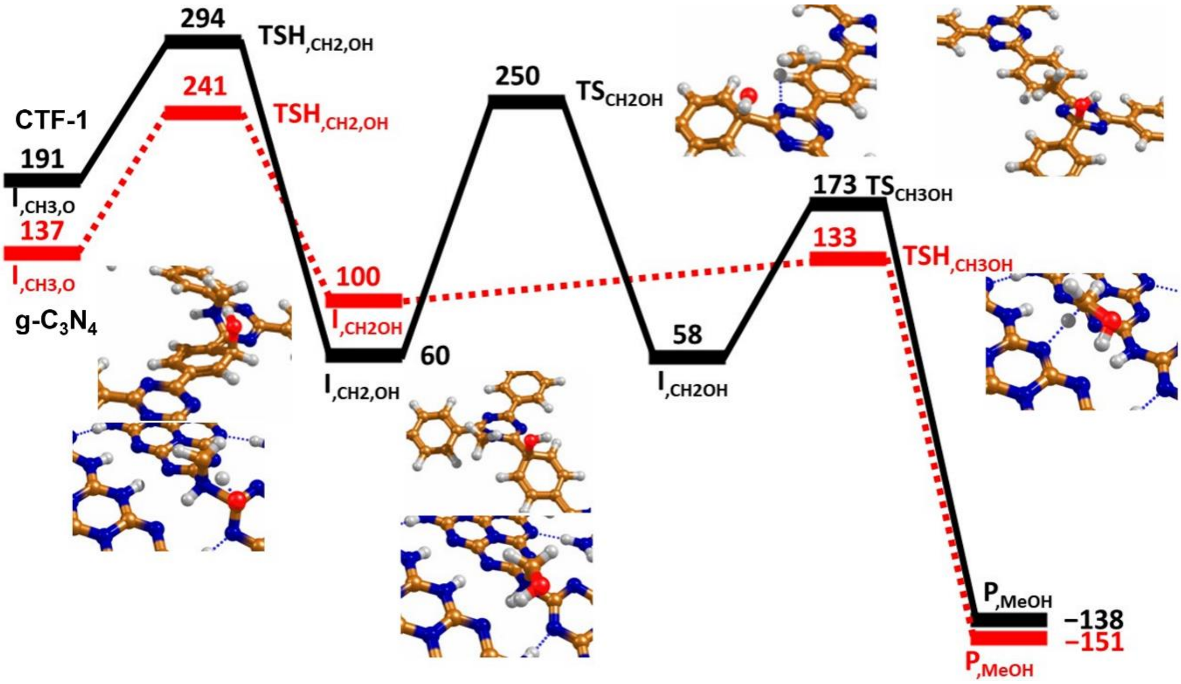

Fig. S42. Reaction energies for methyl radical to methanol conversion on CTF-1 and g-C_3N_4.

Supplementary Note to Fig. S42.

There is no CH_2OH intermediate **(I_{CH2OH})** for g-C_3N_4 because there is a concerted CH_2:OH bond formation affecting proton transfer from the methyl radical to the bond hydroxyl. Importantly, the rate limiting step for CFT-1 is the formation of the same CH_2OH species (**TS_{CH2OH}**), with a barrier of 190 kJ/mol. Therefore, the competing rate limiting barrier(s) for methane conversion to ethanol are lower than those for methane to methanol conversion (see Fig. S26).

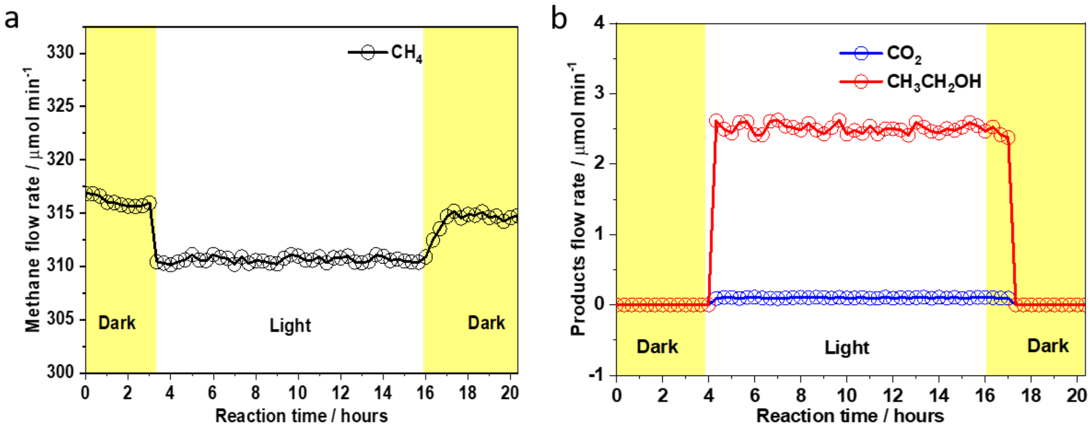

Fig. S43 Stability test of 3 wt.% PtO_x/CTF-1: (a) Methane conversion rate, (b) ethanol and carbon dioxide production rates. Reaction conditions: 100 W 365 nm LED irradiation, GHSV=2000 mL h^{-1}, 16:1 methane (20% methane/argon) to oxygen (water-saturated air, 20% oxygen/nitrogen) flow ratio.

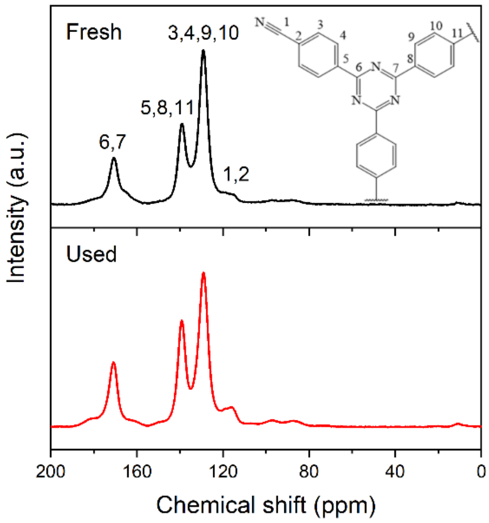

Fig. S44 Solid state ^{13}C NMR spectra of CTF-1 before and after 50 h of photocatalytic methane conversion reaction.

The sample after reaction was dried under vacuum at room temperature to avoid the influence of the adsorbed water. A total of 100 mg catalysts were used for the samples before and after stability test.

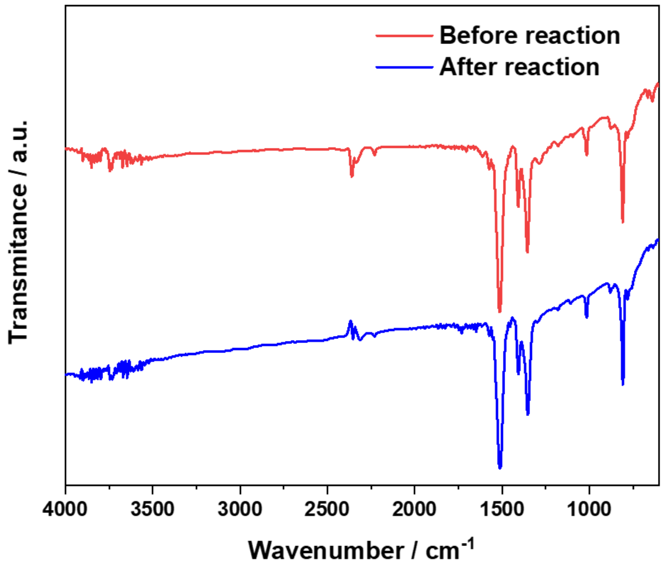

Fig. S45. FTIR spectra before and after 12-hour photocatalytic methane activation reaction.

Supplementary Note to Fig. S43.

The sample after reaction was dried under vacuum at room temperature to avoid the influence of the adsorbed water.

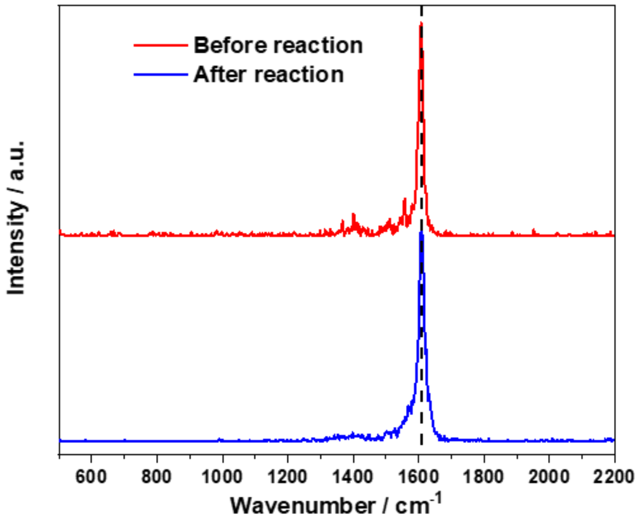

Fig. S46. Raman spectra excited by 325 nm laser in the atmosphere before and after 12-hour photocatalytic methane activation reaction.

Supplementary Note to Fig. S44.

The sample after reaction was dried under vacuum at room temperature.

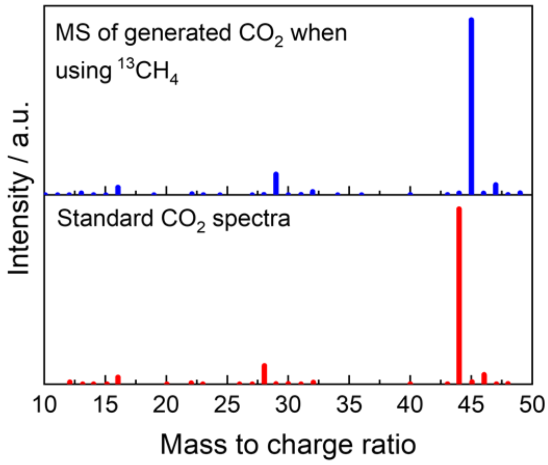

Fig. S47. Mass spectrum of CO_2 obtained by the photocatalytic oxidation of $^{13}CH_4$ (upper panel), and Mass spectrum of standard $^{12}CO_2$ (lower panel).

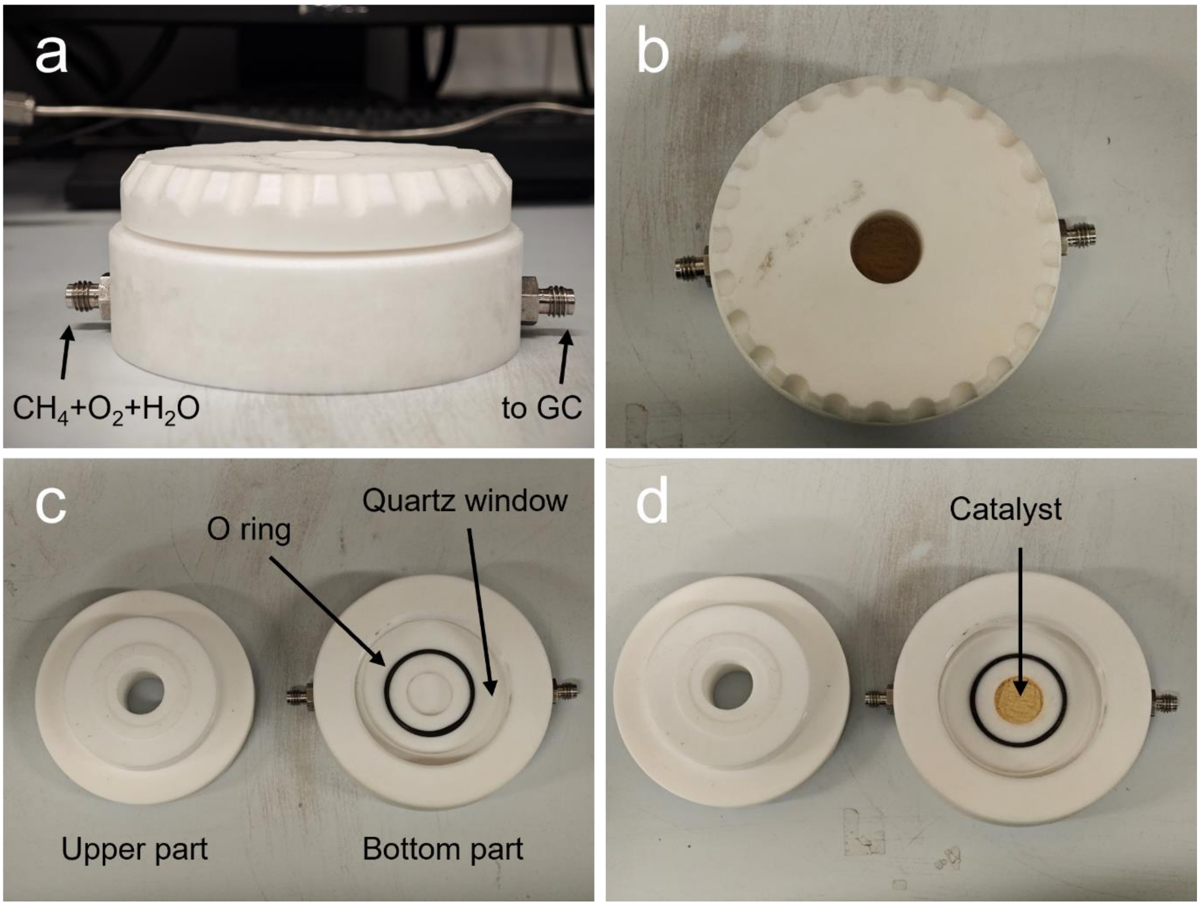

Fig. S48 The flow reactor used for photocatalytic methane conversion. (a) Front view, (b) top view, (c) Upper and bottom parts showing the inner structures, (d) Upper and bottom parts with CTF-1 packed in the reactor.

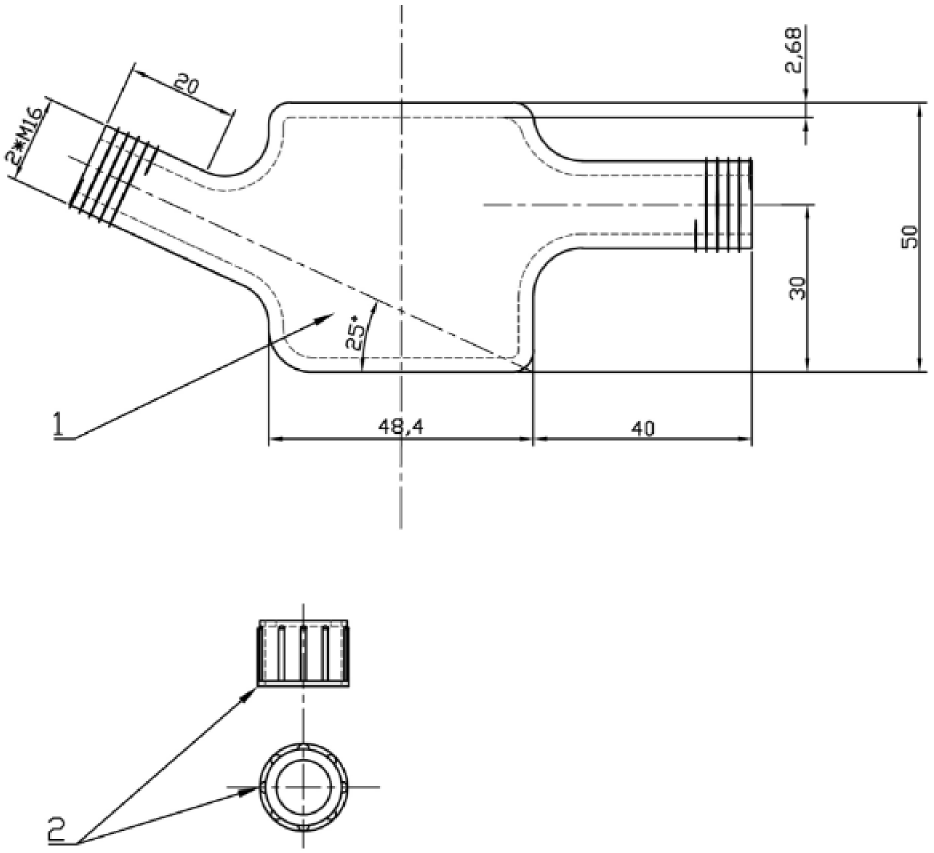

Fig. S49 A diagram showing the batch reactor used for the isotopic labelling experiments. Part 1 shows the reactor, and Part 2 shows the cap.

References

1. Liu, J., Lyu, P., Zhang, Y., Nachtigall, P. & Xu, Y. New Layered Triazine Framework/Exfoliated 2D Polymer with Superior Sodium-Storage Properties. *Advanced Materials* **30**, 1705401 (2018).
2. Kuhn, P., Antonietti, M. & Thomas, A. Porous, covalent triazine-based frameworks prepared by ionothermal synthesis. *Angewandte Chemie International Edition* **47**, 3450–3453 (2008).
3. Weilnboeck, F. *et al.* Photoresist modifications by plasma vacuum ultraviolet radiation: The role of polymer structure and plasma chemistry. *Journal of Vacuum Science & Technology B, Nanotechnology and Microelectronics: Materials, Processing, Measurement, and Phenomena* **28**, 993–1004 (2010).
4. Schwinghammer, K., Hug, S., Mesch, M. B., Senker, J. & Lotsch, B. V. Phenyl-triazine oligomers for light-driven hydrogen evolution. *Energy Environ Sci* **8**, 3345–3353 (2015).
5. Lv, Q. *et al.* Formation of crystalline carbon nitride powder by a mild solvothermal method. *J Mater Chem* **13**, 1241–1243 (2003).
6. Wang, Y. *et al.* Linker-controlled polymeric photocatalyst for highly efficient hydrogen evolution from water. *Energy Environ Sci* **10**, 1643–1651 (2017).
7. Kuecken, S., Schmidt, J., Zhi, L. & Thomas, A. Conversion of amorphous polymer networks to covalent organic frameworks under ionothermal conditions: A facile synthesis route for covalent triazine frameworks. *J Mater Chem A Mater* **3**, 24422–24427 (2015).
8. Mizuno, K., Miyashita, Y., Shindo, Y. & Ogawa, H. NMR and FT-IR studies of hydrogen bonds in ethanol-water mixtures. *Journal of Physical Chemistry* **99**, 3225–3228 (1995).
9. Vinayan, B. P. & Ramaprabhu, S. Platinum–TM (TM = Fe, Co) alloy nanoparticles dispersed nitrogen doped (reduced graphene oxide-multiwalled carbon nanotube) hybrid structure cathode electrocatalysts for high performance PEMFC applications. *Nanoscale* **5**, 5109 (2013).
10. Park, K. C. *et al.* Carbon-supported Pt-Ru nanoparticles prepared in glyoxylate-reduction system promoting precursor-support interaction. *J Mater Chem* **20**, 5345–5354 (2010).
11. Zhou, Y., Zhang, L. & Wang, W. Direct functionalization of methane into ethanol over copper modified polymeric carbon nitride via photocatalysis. *Nat Commun* **10**, 506 (2019).
12. Du, J. *et al.* Evoked Methane Photocatalytic Conversion to C2 Oxygenates over Ceria with Oxygen Vacancy. *Catalysts* **10**, 196 (2020).
13. Du, X. *et al.* Efficient Photocatalytic Conversion of Methane into Ethanol over P-Doped g-C_3N_4 under Ambient Conditions. *Energy & Fuels* **36**, 3929–3937 (2022).
14. Yang, Z. *et al.* Efficient photocatalytic conversion of CH_4 into ethanol with O_2 over nitrogen vacancy-rich carbon nitride at room temperature. *Chemical Communications* **57**, 871–874 (2021).
15. He, C. *et al.* Photocatalytic Conversion of Methane to Ethanol at a Three-Phase Interface with Concentration-Matched Hydroxyl and Methyl Radicals. *J Am Chem Soc* **146**, 11968–11977 (2024).
16. Scoot, S. L. A Matter of Life(time) and Death. *ACS Catal* **8**, 8597–8599 (2018).
17. Schüth, F., Ward, M. D. & Buriak, J. M. Common Pitfalls of Catalysis Manuscripts Submitted to *Chemistry of Materials*. *Chemistry of Materials* **30**, 3599–3600 (2018).
18. Takata, T. *et al.* Photocatalytic water splitting with a quantum efficiency of almost unity. *Nature* **581**, 411–414 (2020).
19. Zhang, Y. *et al.* Internal quantum efficiency higher than 100% achieved by combining doping and quantum effects for photocatalytic overall water splitting. *Nat Energy* **8**, 504–514 (2023).

20. Qureshi, M. & Takanabe, K. Insights on Measuring and Reporting Heterogeneous Photocatalysis: Efficiency Definitions and Setup Examples. *Chemistry of Materials* **29**, 158–167 (2017).
21. Wang, Z., Li, C. & Domen, K. Recent developments in heterogeneous photocatalysts for solar-driven overall water splitting. *Chem Soc Rev* **48**, 2109–2125 (2019).
22. Li, X., Wang, C. & Tang, J. Methane transformation by photocatalysis. *Nat Rev Mater* **7**, 617–632 (2022).
23. Meng, X. *et al.* Direct Methane Conversion under Mild Condition by Thermo-, Electro-, or Photocatalysis. *Chem* **5**, 2296–2325 (2019).
